# Global, regional and national burden of inflammatory bowel disease in females from 1990 to 2021: an analysis of the global burden of disease study 2021

**DOI:** 10.3389/fgwh.2025.1580451

**Published:** 2025-05-29

**Authors:** Jingyi Peng, Yuan Yuan, Jie Zhang, Yang Ding, Xingxing He

**Affiliations:** ^1^ Department of Gastroenterology, Zhongnan Hospital of Wuhan University, Wuhan, China; ^2^ Hubei Provincial Clinical Research Center for Intestinal and Colorectal Diseases, Hubei Key Laboratory of Intestinal and Colorectal Diseases, Wuhan, China

**Keywords:** inflammatory bowel disease, females, disease burden, sociodemographic index, DALYs

## Abstract

**Background & aims:**

The global incidence of inflammatory bowel disease (IBD) has markedly increased over past several decades. Gender-specific differences have been observed in the epidemiology, manifestation, and prognosis of IBD. Given these distinctions, a focused analysis of the latest epidemiological trends in female patients is essential for advancing targeted healthcare.

**Methods:**

A comprehensive analysis of IBD incidence, mortality, and disability-adjusted life years (DALYs) in females was performed using data from the Global Burden of Disease (GBD) study from 1990 to 2021, with stratifications by age, region, country, and sociodemographic index (SDI).

**Results:**

In 2021, approximately 187,134 females were diagnosed with IBD globally. Incidence rates were highest among females aged 30–60, with disease burden increasing significantly with age in older populations. Geographically, High-Income North America had the highest IBD burden in females in 2021, while Australasia experienced the most significant increase from 1990 to 2021 (estimated average percentage changes (EAPC) = 1.13, 95% CI 0.8–1.46). Nationally, 75 countries and territories showed upward trends in the age-standardized DALYs (disability-adjusted life years) rate (ASDR), with the steepest rise observed in Mauritius (EAPC = 2.28, 95% CI 0.82–3.76). DALYs due to IBD in females also increased across all SDI regions, showing a positive correlation between SDI and ASDR.

**Conclusions:**

The global burden of IBD in females has significantly risen from 1990 to 2021, with marked age, regional and SDI-based differences. Incidence rates are higher in high-income regions in Europe and North America, with the sharpest increases observed in East Asia, highlighting the need for age and region-specific IBD management strategies.

## Highlights

• In 2021, approximately 187,134 females were diagnosed with IBD globally. Incidence rates were highest among females aged 30–60, with disease burden increasing significantly with age in older populations.
• High-Income North America had the highest IBD burden in females in 2021.
• 75 countries and territories showed upward trends in ASDR, 182 countries and territories exhibited a decline in ASDR.
• A positive correlation was observed between SDI and the age-standardized DALY rate.

## Introduction

Inflammatory bowel disease (IBD) is a kind of chronic, progressive, immune-mediated gastrointestinal diseases which mainly includes Crohn's disease (CD) and ulcerative colitis (UC), accompanied with diverse clinical manifestations and long-term complications (1, 2). It was estimated that the global incident cases of IBD remained a rising trend over past 30 years (3), which has become global diseases and placed an increasing burden to health management.

Gender-specific differences in IBD epidemiology are complex, varying by geographic region and age (4). For instance, an epidemiology study in 2019 predicted that the incidence rate in male patients was higher than in female patients with IBD globally, while the years lived with disabilities is higher in female patients (5). Another research focus on the United States showed that the prevalence ratio among males under 20 was higher than females, with no significant differences in other age range (6). Studies have shown that female IBD patients, especially those with Crohn's disease, tend to have more severe clinical symptoms and disabilities than their male counterparts, with comorbidities showing strong gender-specific manifestations (7, 8). Besides, female patients with IBD are more susceptible to depression and anxiety (9, 10). Therefore, it is essential to recognize the gender-specific difference of IBD patients in multiple aspects. Analyzing the epidemiological trends among female IBD patients can clarify gender differences in the disease and provide a scientific foundation for developing personalized medical care and public health policies, ultimately improving the health and quality of life for female IBD patients.

This study provides an overview of IBD epidemiological trends in females from 1990 to 2021 using data from the Global Burden of Disease (GBD) study at global, regional, and national levels. These data offer valuable insights into the epidemiological characteristics of IBD in females and support future healthcare policy development.

## Methods

### Study data

The data presented in the article originated from the Global Burden and Disease Study (GBD) 2021 (https://vizhub.healthdata.org/gbd-results/), which had calculated the disease burden on a global scale, across seven super-regions, 21 regions, 204 countries and territories (including subnational locations within 21 countries), and 811 subnational locations, for the period from 1990 to 2021 (11). For the GBD 2021 evaluation, Inflammatory Bowel Disease (IBD) was identified using the International Classification of Diseases, 10th Revision (ICD-10 codes: K50-K51.319, K51.5-K52, K52.8-K52.9) (12). For the models of ulcerative colitis and Crohn's disease, the GBD database utilized MR-BRT analysis to adjust non-reference data points. Using the DisMod-MR 2.1 model, estimates were generated by age, sex, year, and country. The settings featured an incidence rate of 0 for individuals aged 0–2 years and 0.00025 for those aged 80–100 years, along with a remission and excess mortality rate of 0.2 across all ages. The minimum coefficient of variation was set to 0.8 at various levels. Predictive covariates included the sociodemographic index for incidence and the healthcare access and quality index for EMR (11).

To better understand the relationship between economic development and health outcomes, the GBD study used the World Bank's income classification system to categorize countries based on their level of economic development. Accordingly, this system grouped nations into four categories according to per capita Gross National Income (GNI). These included low-income economies (GNI per capita ≤$1,135), lower-middle-income economies ($1,136–$4,465), upper-middle-income economies ($4,466–$13,845), and high-income economies (GNI per capita ≥$13,846). In this way, the classification provided a structured framework for comparing health indicators across different economic contexts (13).

As a broad indicator of social and demographic development that was strongly linked to health outcomes, the SDI was determined by calculating the geometric mean of three factors: the total fertility rate for those under 25, the average years of schooling for individuals aged 15 and older, and the lagged distribution index of per capita income, which ranged from 0 to 1 (5, 11).

### Statistical analysis

Age-standardized rates (ASR) were utilized to compare mortality and DALY rates across countries with varying age structures and demographic profiles. To assess trends in the ASRs of prevalence, deaths, and DALYs over a specific period, estimated average percentage changes (EAPCs) were computed. A linear relationship was assumed between the natural logarithm of the rate and time, expressed as y = *α* + βx + *ε*, where x = calendar year, y = ln(rate), and *ε* = error term. The EAPC was calculated as 100 ×  (e^∧^ β − 1) with a 95% confidence interval (CI). The ASR was considered to have increased if both the EAPC and the lower 95% CI limit were above 0, decreased if both were below 0, and remained stable otherwise (12, 14). The association between ASR and SDI was visualized using a Gaussian process regression model with Loess smoothing and was assessed via Spearman rank correlation tests (14).

All rates were presented per 100,000 population. Using R version 4.3.3, data processing, analysis, and visualization were conducted.

## Results

### Global burden of inflammatory bowel disease (IBD) in females

From 1990 to 2021, the global incidence of IBD in females rose from 98,746.19 (95% UI, 87,064.04–115,384.75) to 187,134.64 (95% UI, 163,469.2–217,222.7) (Supplementary Table S1). From 1990 to 2021, the incidence of the age-standardized incidence rate (ASIR) of IBD in females increased significantly, with an upward trend (EAPC = 0.31), as shown in Supplementary Table S1. The absolute number of IBD-related deaths among females also increased, while the age-standardized mortality rate (ASMR) declined, with a significant downward trend (EAPC = –0.31) (Supplementary Table S2). In terms of disability-adjusted life years (DALYs), the total DALYs in females increased, but the age-standardized DALY rate decreased from 1990 to 2021 (EAPC = –0.48), as presented in Table 1.

**Table 1:** Global and regional DALYs of inflammatory bowel disease among females in 1990 and 2021, and EAPC of ASDR from 1990 to 2021.

Location	DALY number in 1990	ASDR in 1990 (per 100,000)	DALY number in 2021	ASDR in 2021 (per 100,000)	EAPC, 1990–2021
Global	486,463.38 (374,188.99–601,528.52)	21.18 (16.65–25.88)	776,214.74 (661,484.54–929,322.42)	17.75 (15.08–21.23)	−0.48 (−0.58 to 0.39)
SDI					
High SDI	189,014.8 (157,334.8–228,636.78)	34.04 (27.89–41.7)	326,576.34 (272,831.07–386,042.8)	35.75 (28.88–43.78)	0.36 (0.14–0.57)
High-middle SDI	95,976.84 (78,544.74–114,772.01)	17.96 (14.59–21.55)	115,035.1 (95,270.15–139,167.53)	12.23 (10.08–14.81)	−1.34 (−1.42 to 1.27)
Middle SDI	103,134.36 (57,875.16–138,381.09)	15.54 (9.03–20.38)	132,797.1 (107,737.65–161,176.66)	9.86 (7.98–11.96)	−1.52 (−1.58 to 1.46)
Low-middle SDI	67,770.45 (43,102.6–96,676.89)	16.56 (10.96–22.96)	129,714.8 (102,628.93–167,136.7)	14.96 (11.86–19.46)	−0.28 (−0.33 to 0.24)
Low SDI	30,091.59 (16,756.21–48,673.43)	17.16 (10.47–25.82)	71,466.48 (48,329.09–90,144.01)	16.63 (12.04–20.77)	−0.11 (−0.16 to 0.05)
Region					
Andean Latin America	2,214.25 (1,248.85–3,610.58)	12.33 (7.77–18.26)	1,892.3 (1,445.34–2,498.43)	5.81 (4.44–7.65)	−2.62 (−2.95 to 2.29)
Australasia	4,189.85 (3,030.76–5,581.28)	36.14 (25.79–48.35)	10,235.59 (8,006.55–12,904.16)	44.35 (33.36–57.46)	1.13 (0.8–1.46)
Caribbean	3,193.4 (2,459.71–4,136.6)	20.73 (16.3–26.21)	4,073.09 (2,985.43–5,542.98)	15.52 (11.29–21.46)	−1 (−1.14 to 0.86)
Central Asia	6,068.99 (4,863.56–7,368.73)	18.73 (15.35–22.63)	8,127.94 (6,651.38–9,846.42)	16.46 (13.52–19.82)	−0.68 (−0.85 to 0.51)
Central Europe	15,399.21 (12,825.51–18,410.82)	20.9 (17.33–25.1)	17,751.81 (14,892.26–21,396.63)	19.51 (15.89–23.82)	0.03 (−0.2 to 0.27)
Central Latin America	5,648.92 (5,286.43–6,026.48)	9.73 (9.24–10.29)	12,517.94 (11,112.66–14,175.2)	9.2 (8.18–10.41)	0.41 (0.17–0.65)
Central Sub-Saharan Africa	1,765.08 (912.56–2,763.1)	9.55 (5.47–13.85)	4,083.18 (2,768.58–5,803.53)	8.81 (6.06–12.43)	−0.24 (−0.31 to 0.18)
East Asia	88,442.97 (41,080.31–129,371.33)	19.13 (9.32–27.4)	61,467.22 (44,124.64–94,380.4)	6.33 (4.54–9.52)	−3.71 (−3.97 to 3.44)
Eastern Europe	30,900.67 (26,883.65–35,315.64)	19.86 (17.15–22.82)	31,572.18 (27,550.62–35,633.29)	17.87 (15.52–20.48)	−0.82 (−1.28 to 0.36)
Eastern Sub-Saharan Africa	6,098.03 (3,445.6–10,243.76)	10.62 (6.9–15.09)	13,907.66 (9,524–18,042.89)	10.17 (7.13–13.48)	−0.17 (−0.22 to 0.13)
High-income Asia Pacific	11,863.41 (9,056.61–14,476.7)	11.6 (8.81–14.18)	11,965.15 (8,933.03–15,845.02)	7.27 (5.25–9.58)	−1.32 (−1.53 to 1.11)
High-income North America	77,819.15 (62,240.15–97,094.02)	44.27 (34.64–55.91)	146,016.07 (121,252.72–174,213.04)	51.06 (41.65–62.53)	0.66 (0.45–0.88)
North Africa and Middle East	15,875.75 (11,076.6–24,176.73)	13.34 (9.85–19.03)	32,414 (24,959.68–43,093.93)	12.08 (9.36–16.21)	−0.16 (−0.23 to 0.1)
Oceania	55.78 (38.37–81.92)	2.66 (1.85–3.48)	108.54 (79.19–143.51)	2.2 (1.59–2.88)	−0.71 (−0.79 to 0.63)
South Asia	69,101.56 (42,974.23–102,925.82)	18.86 (12.27–26.92)	135,289.69 (100,820.18–186,761.42)	15.99 (11.97–22.05)	−0.51 (−0.58 to 0.44)
Southeast Asia	13,399.06 (5,921.23–19,569.15)	8.24 (3.73–12.31)	17,250.1 (11,606.93–21,877.8)	5.02 (3.33–6.41)	−1.9 (−2.05 to 1.76)
Southern Latin America	3,994.09 (3,257.52–4,851.67)	15.96 (13–19.49)	5,826.86 (4,663.34–7,330.7)	13.72 (10.75–17.53)	−0.43 (−0.55 to 0.3)
Southern Sub-Saharan Africa	2,404.92 (1,674.02–3,204.65)	11.66 (8.26–14.76)	4,265.63 (3,127.3–5,650.51)	11.61 (8.47–15.32)	0.3 (−0.04 to 0.64)
Tropical Latin America201	9,308.09 (8,771.31–10,054.08)	15.34 (14.46–16.51)	23,127.14 (20,915.42–25,438.82)	17.02 (15.43–18.7)	0.39 (0.16–0.61)
Western Europe	100,241.71 (84,173.76–119,245.37)	37.83 (30.67–46.2)	175,058.02 (148,228.85–206,004.47)	42.72 (34.53–52.4)	0.6 (0.34–0.86)
Western Sub-Saharan Africa	18,478.49 (10,475.89–25,806.74)	23.35 (13.74–32.31)	59,264.65 (31,640.09–82,832.35)	26.96 (14.86–37.59)	0.52 (0.47–0.58)

**Figure 1:**
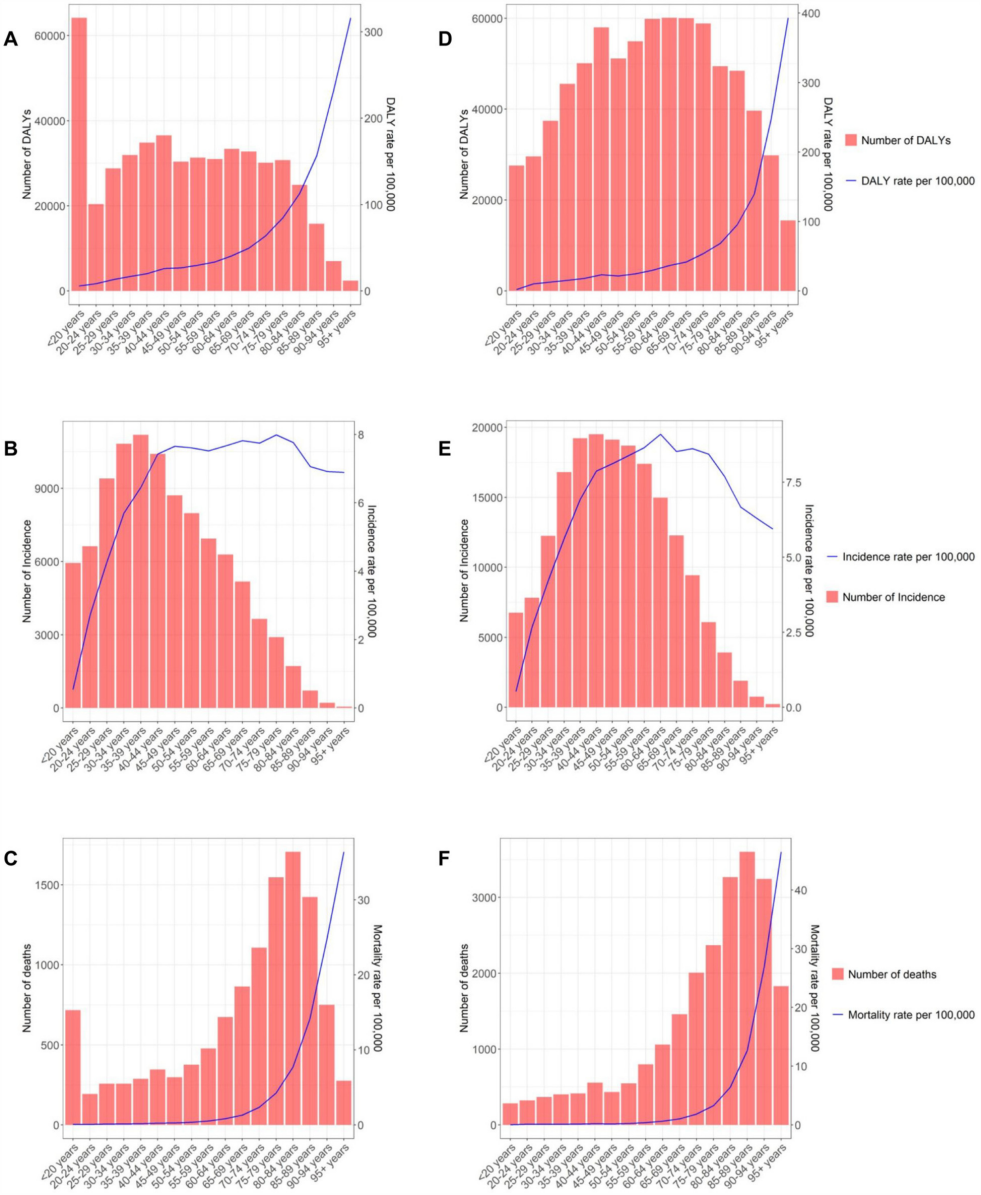
Global incidence **(A)**, deaths **(B)** and DALYs **(C)** rates of IBD for females in different age groups in 1990 and global incidence **(D)**, deaths **(E)** and DALYs **(F)** rates of IBD for females in different age groups in 2021. ASIR, age-standardized incidence rate; ASMR, age-standardized deaths rate; ASDR, age-standardized DALYs (disability-adjusted life years) rate; IBD, inflammatory bowel disease.

Across age groups, the distribution of incidence cases remained largely consistent, with most cases concentrated in the 25–70-year age range (Figure 1A). The EAPC of global incidence in females showed an increasing trend in the 20–80-year age group, whereas other age groups displayed a declining trend (Supplementary Figure S1A). Mortality patterns were largely stable, except for a marked decline in the <20 age group by 2021 (Figure 1B; Supplementary Figure S1B). The highest DALY burden shifted from younger (<20) to older age groups (60–64 years) over time, primarily affecting females aged 30–60 (Figure 1C). DALY rates declined in most age groups, particularly among those under 20, with increases observed only in the 20–24 and 90 + groups (Supplementary Figure S1C).

### Geographical region burden of IBD in females

**Figure 2:**
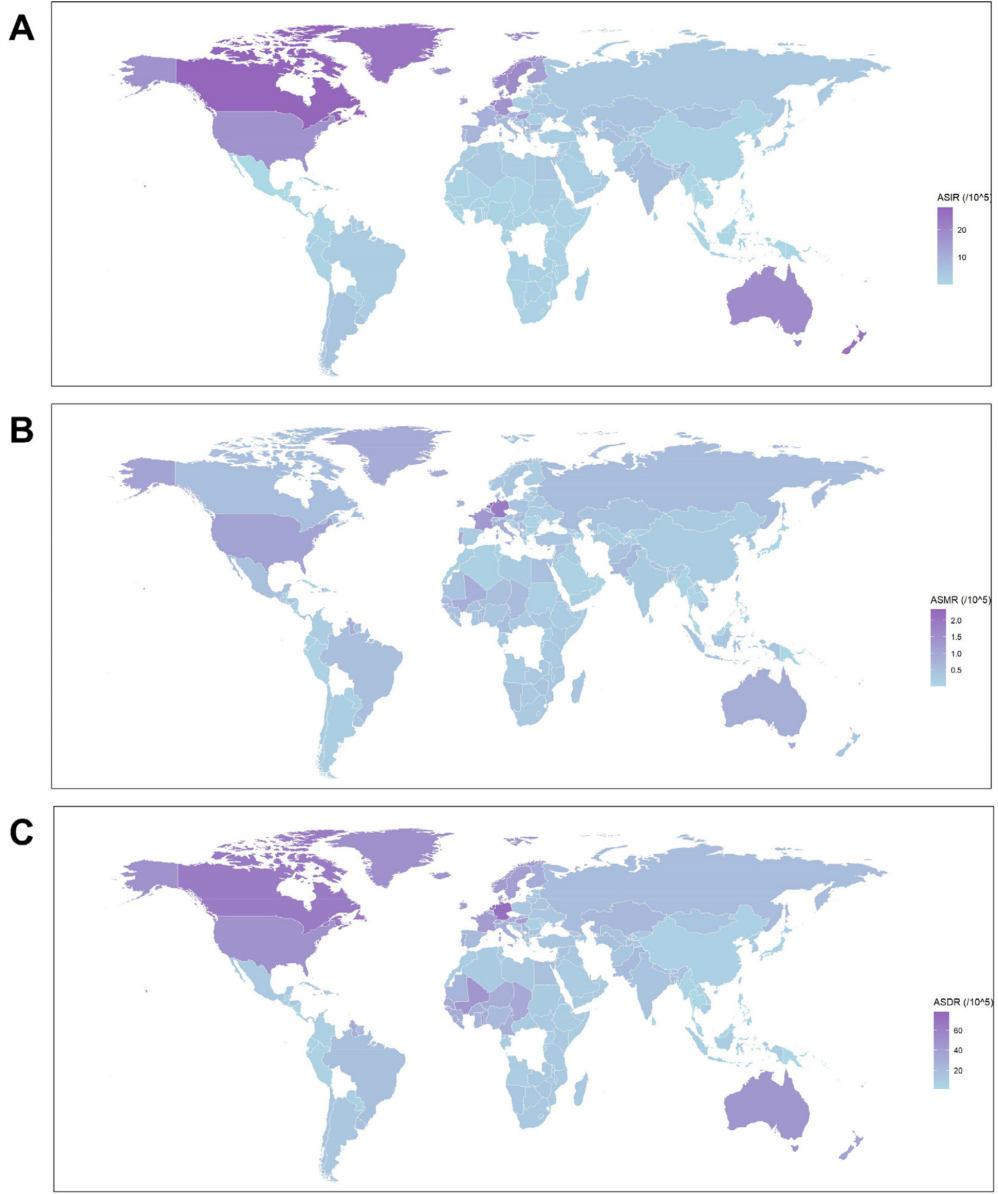
The global distribution of ASIR **(A)**, ASMR **(B)**, and ASDR **(C)** for IBD among females in 2021. ASIR, age-standardized incidence rate; ASMR, age-standardized deaths rate; ASDR, age-standardized DALYs (disability-adjusted life years) rate; IBD, inflammatory bowel disease.

Across all 21 regions, both the incidence and ASIR of IBD in females increased in 2021. High-Income North America, Australasia, and Western and Central Europe showed the highest ASIR, while East Asia experienced the most rapid increase (EAPC = 2.91), as shown in Figure 2A; Supplementary Figure S2A, Supplementary Table S1. In terms of mortality, all regions except High-Income Asia Pacific saw an increase in the number of deaths from IBD in 2021. ASMR increased in 8 regions, with High-Income North America, Western Europe, and Australasia reporting higher ASMR. Australasia showed the highest EAPC for ASMR of 3.82 (95% CI, 2.93 to −4.73) (Figure 2B; Supplementary Figure S2B, Supplementary Table S2). Conversely, ASMR decreased in 13 regions, with High-Income Asia Pacific, East Asia, and Andean Latin America showing the most significant decreases (Supplementary Table S2). Regarding DALYs, 19 regions showed an increase in DALYs in 2021, while only Andean Latin America and East Asia exhibited a decrease. ASDR increased in eight regions (Table 1). The highest ASDR was observed in High-Income North America, Australasia, Western Europe, Western Sub-Saharan Africa, and Central Europe (Figure 2C), with Australasia showing the steepest ASDR increase (EAPC = 1.13) Conversely, 14 regions exhibited a decreasing ASDR, w while East Asia showed the largest decline (EAPC = –3.71) (Supplementary Figure S2C; Table 1**)**.

### Five sociodemographic Index (SDI) regions burden of IBD in females

**Figure 3:**
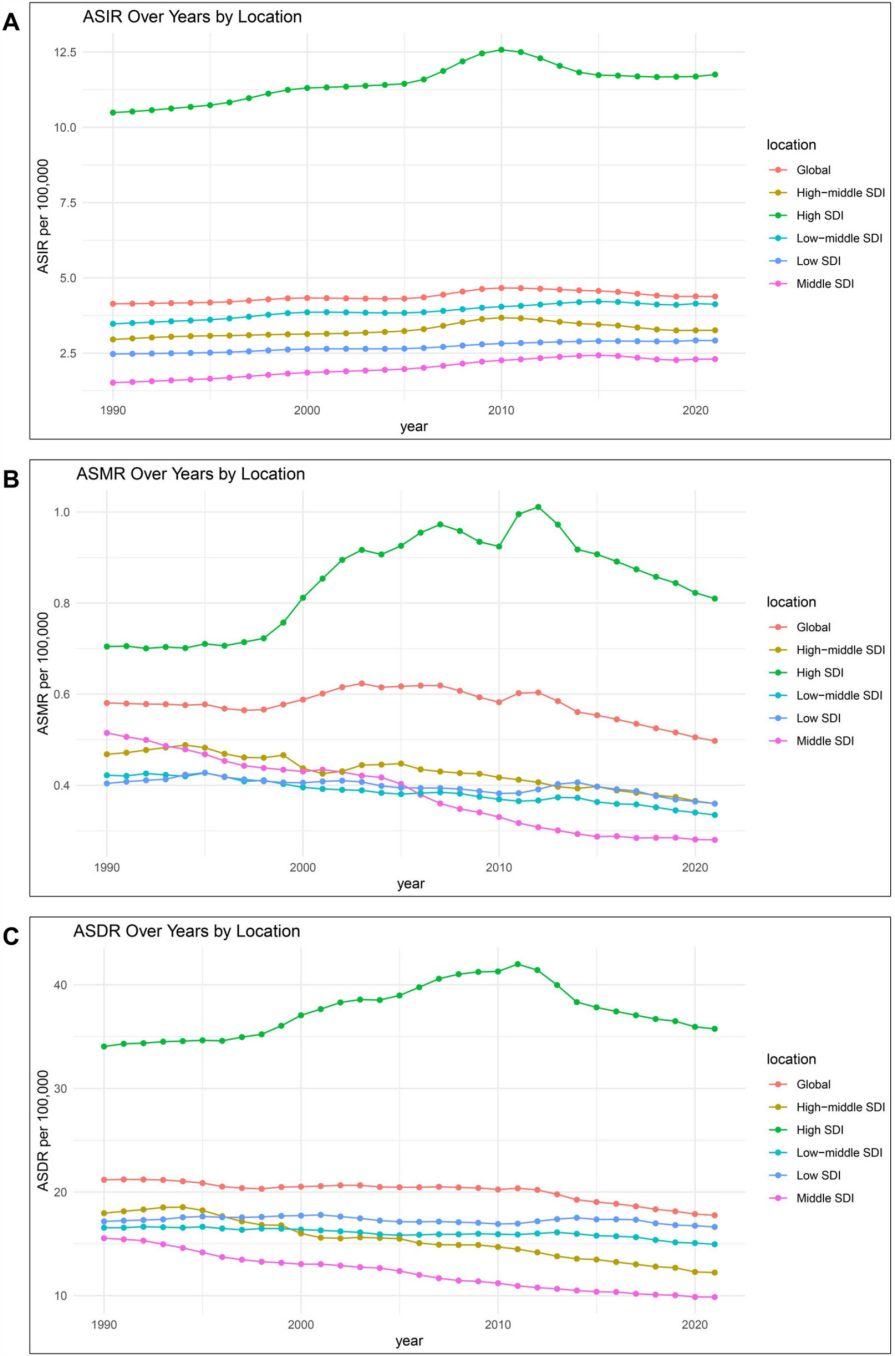
Temporal trends of ASIR **(A)**, ASMR **(B)** and ASDR **(C)** of IBD for females from1990 to 2021 in different SDI regions. ASIR, age-standardized incidence rate; ASMR, age-standardized deaths rate; ASDR, age-standardized DALYs (disability-adjusted life years) rate; IBD, inflammatory bowel disease; SDI, socio-demographic index.

The incidence of IBD in females demonstrated a significant increase across all five SDI regions (Supplementary Table S1). Moreover, compared to 1990, ASIR in each SDI region also rose to varying degrees by 2021 (Supplementary Table S1). Over past 30 years, the ASIR in high-SDI regions remained substantially higher than in other SDI regions, reaching a peak of 12.5 per 100,000 population in 2010 (Figure 3A). In high-SDI regions, the ASMR increased gradually, reaching its peak in 2012 before beginning a slow decline. However, compared to 1990, ASMR still increased overall and surpassed 0.8 per 100,000 population in 2021 (Figure 3B). A decreasing trend in ASMR was observed in other SDI regions, with the most significant reduction occurring in the middle-SDI regions (Figure 3B; Supplementary Table S2). The DALYs attributable to IBD in females showed an upward trend across all SDI regions (Table 1). In high-SDI regions, ASDR exhibited some fluctuations but showed an overall upward trend over past three decades. In contrast, ASDR in high-middle and middle-SDI regions gradually declined, while in low-middle and low-SDI regions, ASDR fluctuated slightly without a clear directional trend (Figure 3C).

### National burden of IBD in females

At the national level, the ASIR, ASDR and ASMR of IBD in females in 2021, along with the EAPC of ASDR, ASIR, and ASMR from 1990 to 2021 were exhibited in Supplementary Table S3; Figure 2. Canada, New Zealand, and Greenland reported the highest ASIRs in 2021. Over past three decades, ASIR increased in 179 countries, with China, Libya, and Taiwan showing the most rapid rises, while 25 countries, including Finland, Saudi Arabia, and Iceland showed declines (Figure 2A; Supplementary Figure S2A). Conversely, 25 countries and territories showed a decline in ASIR, with Finland, Saudi Arabia and Iceland experiencing the most significant decreases (Figure 2A; Supplementary Figure S2A). In terms of ASMR, the highest rates were reported in the Netherlands, Germany and Cyprus, respectively (Figure 2B; Supplementary Table S3). 79 countries and territories exhibited upward trends of ASDR, with the most rapid increases observed in Kuwait, United Arab Emirates and Australia (Supplementary Figure S2B). In contrast, 125 countries and territories exhibited a decrease in ASMR, with the most pronounced reductions in Singapore, Estoniaand South Korea (Supplementary Figure S2B). For ASDR, the highest rates were observed in the Netherlands, Germany wand Canada (Figure 2C; Supplementary Table S3). 75 countries and territories exhibited upward trends of ASDR, with Mauritius, Sierra Leone and Libya showing the most rapid increases (Supplementary Figure S2C). Meanwhile, 182 countries and territories exhibited a decline in ASDR, with the steepest decreases observed in Estonia, China and Bermuda.

### Association between ASIR, ASDR and ASMR with SDI

The association between ASIR, ASDR, and ASMR with SDI in different regions and countries from 1990 to 2021 was shown in Supplementary Figure S3, Supplementary Figure S4. Analysis of the association between ASIR and SDI for IBD in females indicated a moderately strong positive correlation, with ASIR rapidly increasing when SDI ranges from 0.6 to 0.8 and remaining at a high level in regions with SDI > 0.8 (Supplementary Figure S3A). In low SDI regions (e.g., Eastern and Western Sub-Saharan Africa), ASIR remained low. Middle-SDI regions, such as Asia and Latin America, experienced rising IBD incidence, while high-SDI regions like High-Income North America and Australasia showed high ASIR. The ASMR had a weak positive correlation with SDI, with mortality rates relatively low across all SDI levels but slightly elevated in middle to high SDI regions (Supplementary Figure S3B). The correlation between SDI and ASDR is moderate, indicating a statistically significant positive association (Supplementary Figure S3C). ASDR varied more in low and middle SDI regions, reflecting disparities in mortality. Despite high ASDR in regions like High-Income North America and Western Europe, the High-Income Asia Pacific had lower ASDR, indicating SDI is positively correlated with ASMR to some extent, but there are regional differences.

At the national level, a strong positive correlation was observed between SDI and ASIR, with higher rates seen in high SDI countries such as Canada, Germany, and the Netherlands (Supplementary Figure S4A). The correlation between SDI and ASMR was very weak and statistically insignificant, indicating no clear association between SDI and mortality rate for IBD (Supplementary Figure S4B). Minimal variation in ASMR was observed across SDI levels, which suggested that, regardless of a country's socio-demographic status, the mortality rate of IBD in females did not follow a discernible pattern. There was a weak positive correlation between SDI and ASDR, indicating a statistically significant but modest association (Supplementary Figure S4C). The trend line showed a U-shaped curve, suggesting that ASDR was generally lower in countries with moderate SDI levels, while countries with low SDI, like Somalia and Niger, and high SDI countries, such as Canada and Germany, had higher ASDR.

## Discussion

In this study, we systematically analyzed the global epidemiological trends in females with IBD from 1990 to 2021. By comprehensively assessing different age groups, geographic regions, and SDI levels, the study revealed trends in female IBD incidence, mortality, and burden of disease. The data indicated that approximately 187,134 females were diagnosed with IBD worldwide in 2021. The highest incidence occurred among females aged 30–60, with incidence peaking in the 60–64 age group. ASMR and ASDR remain generally stable in the younger and middle-age groups; however, the disease burden sharply increases with age in older adult populations. Given the unique disease manifestations, complication rates, and medication responses in older patients, along with increased susceptibility to comorbidities, debilitation and polypharmacy (15), managing IBD in older adult patients presents multiple challenges. Although disease burden in younger and middle-aged females is significantly lower than in older adults, the potential impact of rising incidence among these groups warrants attention. Furthermore, as active IBD has been associated with increased risks of preterm labor, low gestational weight, and fetal miscarriage (16, 17), the broader health impacts of IBD on young and middle-aged women must be considered, highlighting the importance of addressing their specific healthcare needs. Although newly published studies showed that the incidence of IBD is higher in females than in males before the age of 45 years and lower after the age of 45 years, the number of deaths and DALYS are higher in females than in males at old age, and the difference is not significant in young and middle age (3). However, female patients often face more challenges than males in the management of IBD, including the safety of therapeutic medications, disease management during pregnancy, and mental health issues (10, 18). Studies have also shown that female IBD patients have a higher risk of nutrient deficiencies and anemia than male patients (19, 20). Therefore, in order to reduce the disease burden and improve the quality of life of female IBD patients, enhancing disease awareness through public health education, integration of multidisciplinary health care support, and enhancement of mental health support among female patients is imperative.

Geographic regions and SDI levels significantly influence the epidemiologic profile of IBD in females. The incidence of female patients is notably higher in high-income countries and regions than in low- and middle-income regions, consistent with the trend in recent years (21). This trend is closely related to lifestyle and environmental factors in high-income countries, such as westernized diets (high-fat, low-fiber), lifestyles, and accelerated urbanization, which may lead to the intestinal dysbiosis, thus increasing the risk of IBD (22, 23). Despite this, the increase of ASIR in high SDI regions was lowest than other SDI regions, indicating that high SDI regions have reached the epidemiological stages of compounding prevalence. In this stage, efforts should prioritize managing long-term complications and comorbidities. In contrast, middle SDI regions, exemplified by China, which shows the highest EAPC in ASIR, are in the “acceleration in incidence” stage. This calls for enhanced public health initiatives to monitor changes in environmental and dietary factors. Low SDI regions are in the “emergence” stage, where establishing baseline data on IBD incidence and prevalence should be prioritized (24). Notably, middle SDI regions showed a significant increase in incidence of IBD but a marked decline in DALYs. This phenomenon, similarly observed in studies focusing on IBD epidemiology in the BRICS countries (Brazil, Russia, India, China, and South Africa) (25), suggests that these regions are experiencing rapid economic development and accelerated industrialization, alongside gradual improvements in health policies and systems (26). In low SDI countries, the incidence and DALYs of IBD are low. However, the actual burden may be underestimate (27, 28), likely due to limited medical resources and restricted management capabilities (29). Thus, enhancing healthcare accessibility for females with IBD in low-income countries is essential. This may be achieved by strengthening basic healthcare infrastructure, promoting health education and community support, and encouraging the use of affordable and effective medications and treatments.

Several limitations should be taken into account in our study. This study relies primarily on GBD database data, whose completeness and accuracy vary across countries and regions. High-income countries typically have better access to health care and data monitoring systems, whereas data from low- and middle-income countries may be incomplete or inaccurate, potentially leading to an underestimation of IBD burden in these regions. Second, in this study, IBD was analyzed as a single disease, without separately analyzing its two main types, CD and UC. Two IBD subtypes exhibit multiple differences in clinical manifestations, disease progression, and treatment responses. In women, hormonal and reproductive factors can further modify how these conditions present and progress. Grouping both subtypes together may obscure subtype-specific trends and result in less precise assessments of the overall IBD burden among females. To gain a clearer understanding of the epidemiological patterns in female populations, it is essential for future research to analyze Crohn's disease and ulcerative colitis separately. The EAPC analysis assumes that the logarithm of the rates changes linearly over time. While this method is useful for summarizing overall trends, it may not capture nonlinear patterns, sudden fluctuations, or threshold effects commonly seen in real-world epidemiological or demographic data. Moreover, the validity of EAPC results depends on the quality and completeness of the data. Missing or inconsistent data may affect the reliability of trend estimates. Although we made every effort to ensure data consistency, gaps or differences in reporting standards across regions or time periods may still exist, which could influence the robustness of the findings. Although this study incorporates the effect of SDI levels on the burden of IBD in females, it fails to explore factors such as environment, ethnicity, culture, hormones and differences in the public health system. As a kind of chronic diseases, IBD may be significantly affected by patient lifestyle, physiological stages, cultural background, social support, and healthcare accessibility, all of which could influence the disease burden.

## Data Availability

The original contributions presented in the study are included in the article/Supplementary Material, further inquiries can be directed to the corresponding author.
