# Supplementary material for: Global, regional and national burden of inflammatory bowel disease in females from 1990 to 2021: an analysis of the global burden of disease study 2021
Source: Front Glob Womens Health. 2025 May 29;6:1580451. doi: 10.3389/fgwh.2025.1580451 (PMC12158981; doi:10.3389/fgwh.2025.1580451)
Supplement: Supplementary file 1 [file Table1.docx]

**Supplementary materials**

**Global, regional and national burden of inflammatory bowel disease in females from 1990 to 2021: an analysis of the global burden of disease study 2021**

Jingyi Peng^1,2†^, Yuan Yuan^1,2†^, Jie Zhang ^1,2^, Yang Ding^1,2^, Xingxing He^1,2^*

^1^Department of Gastroenterology, Zhongnan Hospital of Wuhan University, Wuhan, China;

^2^Hubei Provincial Clinical Research Center for Intestinal and Colorectal Diseases, Hubei Key Laboratory of Intestinal and Colorectal Diseases

†Jingyi Peng and Yuan Yuan contributed equally to this work

*Correspondence to:

Xingxing He, Department of Gastroenterology, Zhongnan Hospital of Wuhan University, Wuhan, China, Email: [hexingxing@whu.edu.cn](mailto:hexingxing@whu.edu.cn)

**Supplementary materials’** **information**

Supplementary Figure S1. EAPC of global incidence (A), mortality (B) and DALYs (C) rates of IBD in females in different age groups from 1990 to 2021.

Supplementary Figure S2. Temporal trends of ASIR (A), ASMR (B) and ASDR (C) of IBD from 1990 to 2021 in 204 countries and territories.

Supplementary Figure S3. Correlations between ASIR (A), ASMR (B) and ASDR (C) of IBD and SDI at the regional level.

Supplementary Figure S4. Correlations between ASIR (A), ASMR (B) and ASDR (C) of IBD and SDI at the national level.

Supplementary Table S1. Global and regional incidence of inflammatory bowel disease among females in 1990 and 2021, and EAPC of ASIR from 1990 to 2021.

Supplementary Table S2. Global and regional deaths of inflammatory bowel disease among females in 1990 and 2021, and EAPC of ASMR from 1990 to 2021.

Supplementary Table S3. National DALYs, incidence, and deaths of inflammatory bowel disease among females in 2021, and EAPC of ASDR, ASIR, and ASMR from 1990 to 2021.


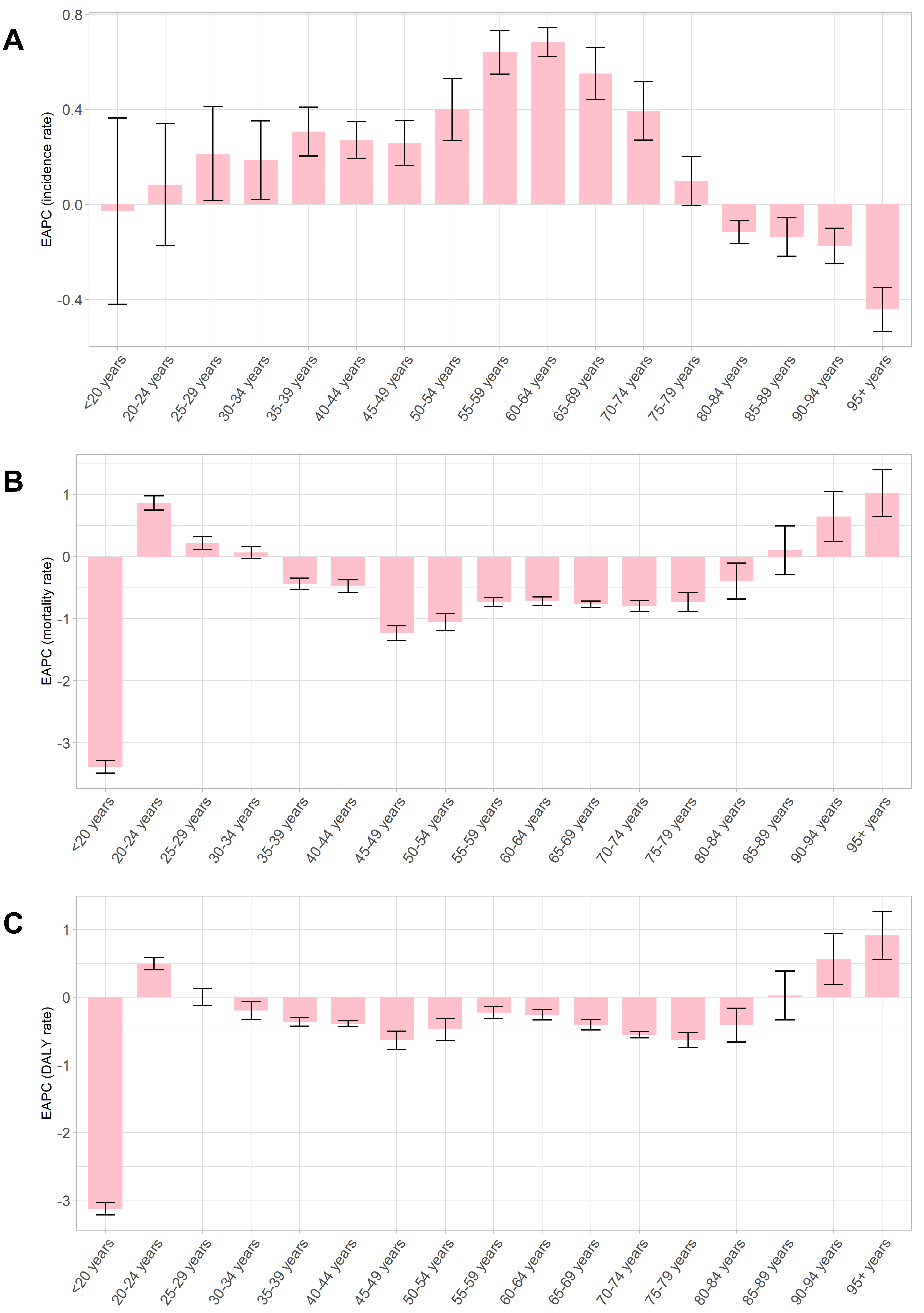


**Figure S1. EAPC of global incidence (A), mortality (B) and DALYs (C) rates of IBD in females in different age groups from 1990 to 2021.** EAPC，estimated annual percentage change; DALYs, disability-adjusted life years; IBD, inflammatory bowel disease.





**Figure S2.** **Temporal trends of ASIR (A), ASMR (B) and ASDR (C) of IBD from 1990 to 2021 in 204 countries and territories.** ASIR, age-standardized incidence rate; ASMR, age-standardized deaths rate; ASDR, age-standardized DALYs (disability-adjusted life years) rate; IBD, inflammatory bowel disease.





**Figure S3.** **Correlations between ASIR (A), ASMR (B) and ASDR (C) of IBD and SDI at the regional level.** Black line represents the expected ASR based on SDIs in all locations. ASIR, age-standardized incidence rate; ASMR, age-standardized deaths rate; ASDR, age-standardized DALYs (disability-adjusted life years) rate; IBD, inflammatory bowel disease; SDI, socio-demographic index.


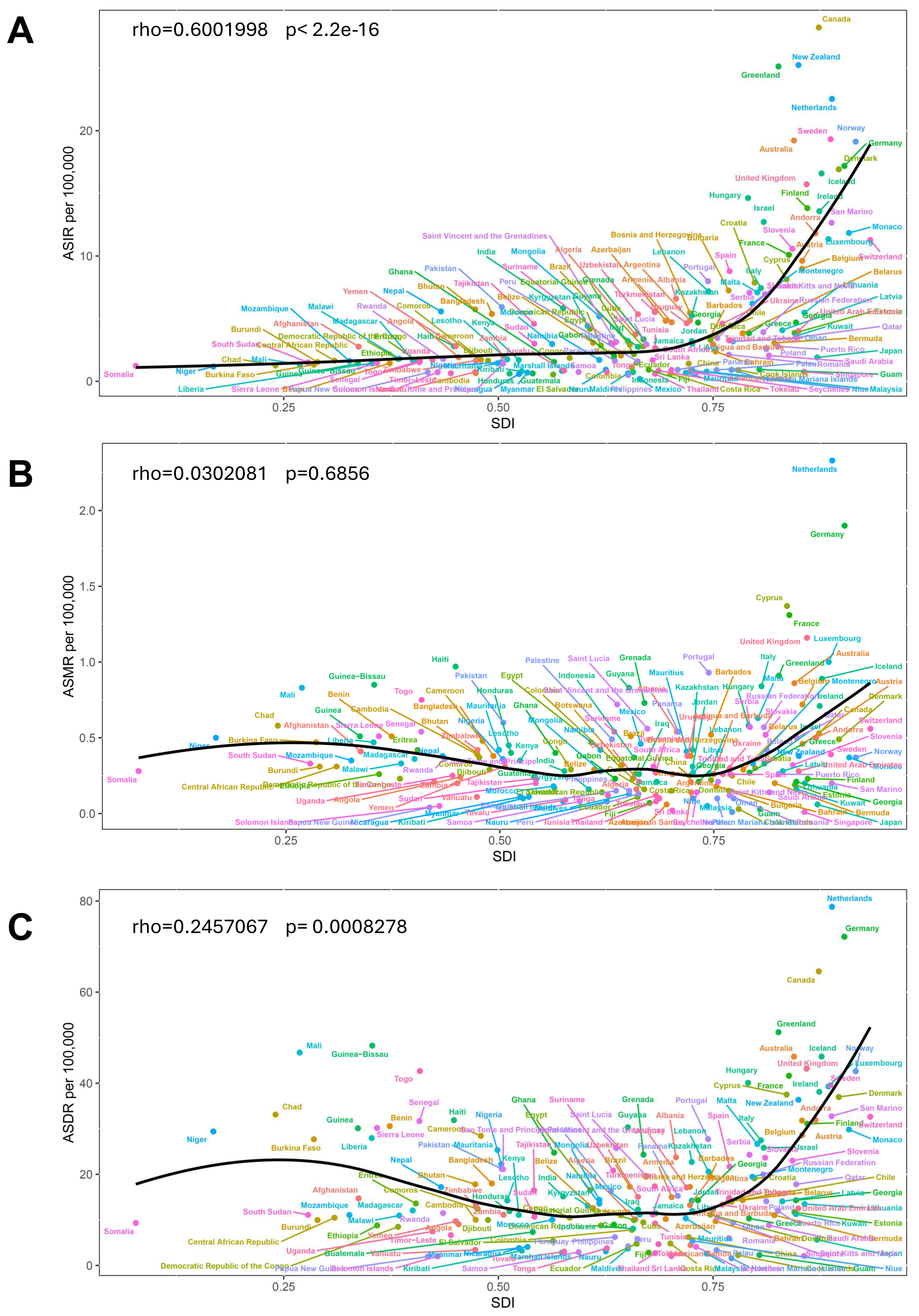


**Figure S4**. **Correlations between ASIR (A), ASMR (B) and ASDR (C) of IBD and SDI at the national level.** Black line represents the expected ASR based on SDI levels in all locations. ASIR, age-standardized incidence rate; ASMR, age-standardized deaths rate; ASDR, age-standardized DALYs (disability-adjusted life years) rate; IBD, inflammatory bowel disease; SDI, socio-demographic index.

**Table S1. Global and regional incidence of inflammatory bowel disease among females in 1990 and 2021, and EAPC of ASIR from 1990 to 2021.**

| Location | incidence number in 1990 | ASIR in 1990 (per 100,000) | incidence number in 2021 | ASIR in 2021 (per 100,000) | EAPC, 1990–2021 |
| --- | --- | --- | --- | --- | --- |
| **Global** | 98746.19 (87064.04-115384.75) | 4.14 (3.66-4.79) | 187134.64 (163469.2-217222.7) | 4.38 (3.82-5.11) | 0.31 (0.21-0.4) |
| **SDI** |  |  |  |  |  |
| High SDI | 52755.22 (46877.85-60896.69) | 10.49 (9.32-12.16) | 80072.97 (70415.71-91395.08) | 11.75 (10.28-13.63) | 0.44 (0.32-0.56) |
| High-middle SDI | 16371.16 (14449.63-19113.18) | 2.95 (2.6-3.45) | 26856.3 (23448.6-31710.19) | 3.26 (2.81-3.87) | 0.49 (0.32-0.66) |
| Middle SDI | 10919.39 (9316.47-13225.13) | 1.52 (1.32-1.82) | 31845.31 (27543.78-38297.65) | 2.3 (1.99-2.76) | 1.59 (1.43-1.75) |
| Low-middle SDI | 14539.04 (12543.97-17420.04) | 3.47 (3.04-4.14) | 36814.37 (31634.54-44648.9) | 4.12 (3.58-4.95) | 0.61 (0.55-0.68) |
| Low SDI | 4061.78 (3461.4-4902.75) | 2.47 (2.14-2.95) | 11404.8 (9752.05-13814.97) | 2.92 (2.52-3.49) | 0.61 (0.57-0.65) |
| **Region** |  |  |  |  |  |
| Andean Latin America | 202.34 (172.65-246.12) | 1.36 (1.17-1.63) | 531.54 (453.38-649.86) | 1.59 (1.35-1.93) | 0.54 (0.48-0.61) |
| Australasia | 2089.89 (1760.14-2525.45) | 18.6 (15.67-22.53) | 3822.4 (3336.53-4467.44) | 20.19 (17.52-23.92) | 0.48 (0.3-0.66) |
| Caribbean | 388.87 (327.03-473.06) | 2.37 (2.02-2.88) | 738.63 (634.03-885.71) | 2.85 (2.43-3.43) | 0.57 (0.52-0.62) |
| Central Asia | 1316.6 (1134.36-1589.77) | 4.35 (3.75-5.22) | 2563.71 (2214.78-3076.63) | 5.09 (4.41-6.07) | 0.51 (0.49-0.53) |
| Central Europe | 3734.25 (3270.18-4380.87) | 5.32 (4.62-6.28) | 4777.18 (4170.92-5523.35) | 6.5 (5.61-7.75) | 0.84 (0.65-1.02) |
| Central Latin America | 353.76 (298.66-438.87) | 0.54 (0.46-0.66) | 743.11 (624.68-919.5) | 0.54 (0.45-0.66) | 0.17 (0.06-0.28) |
| Central Sub-Saharan Africa | 258.98 (221.48-310.5) | 1.36 (1.17-1.62) | 885.57 (750.7-1093.44) | 1.82 (1.56-2.2) | 1.02 (1-1.05) |
| East Asia | 4215.02 (3566.54-5130.33) | 0.74 (0.64-0.9) | 12667.39 (10952.41-15156.24) | 1.39 (1.19-1.67) | 2.91 (2.38-3.43) |
| Eastern Europe | 4916.76 (4244.21-5882) | 3.4 (2.96-4.09) | 6151.26 (5300.3-7362.4) | 4.09 (3.54-4.96) | 0.58 (0.54-0.62) |
| Eastern Sub-Saharan Africa | 831.86 (709.05-1014.71) | 1.34 (1.16-1.6) | 2500.75 (2123.9-3058.24) | 1.66 (1.44-1.97) | 0.81 (0.75-0.87) |
| High-income Asia Pacific | 2022.75 (1767.88-2396.36) | 2.04 (1.78-2.43) | 3004.63 (2615.68-3513.69) | 2.53 (2.18-3.03) | 0.9 (0.39-1.41) |
| High-income North America | 26270.7 (23244.89-30293.32) | 16.19 (14.34-18.77) | 43494.06 (38499.16-49267.75) | 18.5 (16.25-21.21) | 0.51 (0.4-0.61) |
| North Africa and Middle East | 3424.68 (2917.46-4131.77) | 2.67 (2.3-3.21) | 9353.45 (7913.29-11546.78) | 3.15 (2.69-3.86) | 0.59 (0.55-0.63) |
| Oceania | 15.62 (13.19-19.35) | 0.63 (0.54-0.78) | 43.46 (36.46-53.79) | 0.75 (0.63-0.92) | 0.5 (0.46-0.54) |
| South Asia | 18629.75 (16036.12-22434.84) | 4.83 (4.22-5.75) | 51146.62 (44141.92-61494.65) | 5.81 (5.03-6.95) | 0.69 (0.61-0.77) |
| Southeast Asia | 1142.19 (964.06-1396.55) | 0.56 (0.48-0.68) | 2477.1 (2106.32-3017.41) | 0.66 (0.56-0.8) | 0.49 (0.46-0.53) |
| Southern Latin America | 1100.26 (944.95-1314.28) | 4.43 (3.79-5.31) | 1861.03 (1583.47-2287.58) | 4.73 (4.01-5.82) | 0.2 (0.15-0.25) |
| Southern Sub-Saharan Africa | 330.16 (282.08-401.27) | 1.62 (1.4-1.92) | 673.75 (579.78-810.6) | 1.7 (1.48-2.03) | 0.27 (0.18-0.36) |
| Tropical Latin America | 1185.14 (1011.89-1438.29) | 1.83 (1.59-2.2) | 3910.87 (3375.63-4741.01) | 2.89 (2.5-3.5) | 1.04 (0.59-1.5) |
| Western Europe | 25452.48 (22877.78-29043.16) | 11.48 (10.26-13.18) | 33205.32 (29040.97-38227.29) | 12.63 (10.88-14.89) | 0.33 (0.15-0.5) |
| Western Sub-Saharan Africa | 864.13 (740.32-1046.35) | 1.36 (1.18-1.61) | 2582.8 (2214.59-3121.19) | 1.49 (1.3-1.77) | 0.09 (-0.02-0.21) |

**Table S2. Global and regional deaths of inflammatory bowel disease among females in 1990 and 2021, and EAPC of ASMR from 1990 to 2021.**

| Location | deaths number in 1990 | ASMR in 1990 (per 100,000) | deaths number in 2021 | ASMR in 2021 (per 100,000) | EAPC, 1990–2021 |
| --- | --- | --- | --- | --- | --- |
| **Global** | 11556.85 (8945.58-13617.42) | 0.58 (0.46-0.68) | 22967.86 (19363.87-27270.77) | 0.5 (0.42-0.59) | -0.31 (-0.51--0.11) |
| **SDI** |  |  |  |  |  |
| High SDI | 4779.51 (4287.09-5104.49) | 0.7 (0.64-0.75) | 11667.37 (9549.18-12888.8) | 0.81 (0.69-0.88) | 0.91 (0.56-1.27) |
| High-middle SDI | 2416.37 (2023.66-2759.88) | 0.47 (0.39-0.54) | 4006.91 (3313.86-5267.21) | 0.36 (0.3-0.47) | -0.9 (-0.99--0.81) |
| Middle SDI | 2514 (1326.27-3400.33) | 0.51 (0.27-0.68) | 3599.05 (2588.31-4692.79) | 0.28 (0.2-0.37) | -2.2 (-2.34--2.06) |
| Low-middle SDI | 1293 (696.25-1924.57) | 0.42 (0.24-0.62) | 2455.39 (1852.68-3522.43) | 0.33 (0.26-0.48) | -0.72 (-0.78--0.67) |
| Low SDI | 543.21 (281.65-887.27) | 0.4 (0.23-0.59) | 1220.1 (790.77-1599.49) | 0.36 (0.25-0.49) | -0.36 (-0.45--0.28) |
| **Region** |  |  |  |  |  |
| Andean Latin America | 36.8 (21.48-55.5) | 0.27 (0.18-0.37) | 41.97 (29.74-60.87) | 0.13 (0.1-0.19) | -2.37 (-2.62--2.13) |
| Australasia | 43.02 (38.33-47.58) | 0.32 (0.29-0.35) | 284.26 (224.04-330.2) | 0.79 (0.64-0.91) | 3.82 (2.93-4.73) |
| Caribbean | 74.6 (59.62-95.65) | 0.55 (0.45-0.69) | 92.56 (68.92-127.57) | 0.33 (0.24-0.46) | -1.8 (-1.98--1.61) |
| Central Asia | 86.39 (76.19-96.48) | 0.28 (0.25-0.31) | 115.23 (98.14-134.82) | 0.25 (0.22-0.29) | -0.91 (-1.22--0.59) |
| Central Europe | 309.57 (284.09-352.04) | 0.39 (0.36-0.44) | 498.37 (445.08-559.74) | 0.38 (0.34-0.42) | 0.24 (-0.11-0.59) |
| Central Latin America | 154.15 (146.4-160.46) | 0.35 (0.33-0.37) | 441.59 (388.45-496.12) | 0.33 (0.29-0.37) | 0.41 (0.15-0.68) |
| Central Sub-Saharan Africa | 30.21 (14.04-48.42) | 0.25 (0.13-0.4) | 74.25 (43.87-112.2) | 0.24 (0.14-0.4) | -0.08 (-0.11--0.06) |
| East Asia | 2523.02 (1236.09-3517.55) | 0.75 (0.38-1.02) | 2658.19 (1734.94-4522.29) | 0.26 (0.17-0.44) | -3.87 (-4.23--3.5) |
| Eastern Europe | 864.98 (794.36-956.25) | 0.5 (0.46-0.55) | 1024.35 (914.8-1126.22) | 0.47 (0.42-0.52) | -0.85 (-1.67--0.02) |
| Eastern Sub-Saharan Africa | 125.04 (71.67-202.02) | 0.32 (0.2-0.47) | 282.43 (180.3-391.03) | 0.3 (0.19-0.46) | -0.19 (-0.25--0.13) |
| High-income Asia Pacific | 343 (240.3-424.4) | 0.33 (0.23-0.41) | 320.35 (210.14-529.54) | 0.09 (0.06-0.14) | -4.44 (-4.81--4.06) |
| High-income North America | 1506.69 (1317.51-1604.56) | 0.68 (0.6-0.71) | 4018.56 (3382.05-4352.6) | 1.02 (0.89-1.1) | 1.71 (1.28-2.14) |
| North Africa and Middle East | 263.85 (179.56-424.8) | 0.31 (0.21-0.48) | 518.7 (374.9-806.71) | 0.26 (0.19-0.4) | -0.34 (-0.5--0.18) |
| Oceania | 0.81 (0.44-1.26) | 0.07 (0.04-0.09) | 1.62 (0.93-2.29) | 0.05 (0.03-0.08) | -0.63 (-0.7--0.56) |
| South Asia | 1221.49 (606.25-1952.12) | 0.46 (0.24-0.72) | 2216.12 (1511.18-3568.26) | 0.31 (0.22-0.5) | -1.29 (-1.4--1.18) |
| Southeast Asia | 389.55 (149.07-620.37) | 0.32 (0.12-0.52) | 557.72 (330-755.32) | 0.18 (0.11-0.25) | -2.15 (-2.32--1.98) |
| Southern Latin America | 83 (75.4-89.57) | 0.33 (0.3-0.36) | 118.82 (105.25-129.06) | 0.23 (0.21-0.25) | -1 (-1.28--0.73) |
| Southern Sub-Saharan Africa | 52.35 (32.53-69.11) | 0.32 (0.19-0.43) | 104.36 (67.46-146.02) | 0.33 (0.21-0.46) | 0.46 (0.07-0.86) |
| Tropical Latin America | 231.23 (219.03-244.92) | 0.46 (0.43-0.49) | 708.36 (630.44-763.4) | 0.5 (0.45-0.54) | 0.45 (0.16-0.75) |
| Western Europe | 2918.3 (2602.03-3102.74) | 0.79 (0.71-0.84) | 7937.44 (6370.45-8896.41) | 1.1 (0.92-1.21) | 1.73 (1.31-2.15) |
| Western Sub-Saharan Africa | 298.8 (161.73-427.56) | 0.39 (0.22-0.56) | 952.61 (484.42-1371.23) | 0.45 (0.23-0.64) | 0.47 (0.4-0.53) |

**Table S3. National DALYs, incidence, and deaths of inflammatory bowel disease among females in 2021, and EAPC of ASDR, ASIR, and ASMR from 1990 to 2021.**

|  | **DALYs of inflammatory bowel disease** | | **incidence of inflammatory bowel disease** | | **deaths of inflammatory bowel disease** | |
| --- | --- | --- | --- | --- | --- | --- |
| **location** | **ASDR in 2021 (per 100,000)** | **EAPC, 1990-2021** | **ASIR in 2021 (per 100,000)** | **EAPC, 1990-2021** | **ASMR in 2021 (per 100,000)** | **EAPC, 1990-2021** |
| Afghanistan | 14.72 (6.75-34.38) | 0.37 (0.22-0.51) | 2.77 (2.33-3.44) | 0.59 (0.52-0.66) | 0.41 (0.14-0.98) | 0.41 (0.27-0.56) |
| Albania | 23.74 (16.79-33.41) | -1.18 (-1.33--1.02) | 6.59 (5.61-7.92) | 0.93 (0.84-1.03) | 0.54 (0.29-0.95) | -1.33 (-1.50--1.16) |
| Algeria | 10.11 (6.57-15.79) | 0.34 (0.18-0.49) | 3.74 (3.09-4.63) | 0.77 (0.70-0.84) | 0.16 (0.08-0.39) | 0.46 (0.18-0.75) |
| American Samoa | 4.46 (3.09-6.25) | 1.44 (0.92-1.98) | 0.84 (0.72-1.02) | 0.42 (0.37-0.46) | 0.14 (0.08-0.23) | 2.24 (1.41-3.08) |
| Andorra | 31.93 (22.87-43.22) | -0.14 (-0.19--0.09) | 11.81 (9.95-14.83) | 0.13 (0.09-0.17) | 0.50 (0.26-0.80) | -0.58 (-0.76--0.40) |
| Angola | 9.04 (5.17-13.77) | -0.23 (-0.31--0.14) | 1.92 (1.64-2.31) | 0.90 (0.85-0.95) | 0.24 (0.12-0.41) | -0.45 (-0.53--0.37) |
| Antigua and Barbuda | 12.22 (9.94-15.04) | -1.79 (-2.03--1.56) | 2.96 (2.50-3.66) | 0.59 (0.53-0.64) | 0.26 (0.24-0.29) | -2.55 (-2.92--2.19) |
| Argentina | 13.25 (10.01-17.15) | -0.26 (-0.37--0.14) | 4.86 (4.10-5.94) | 0.08 (0.05-0.11) | 0.21 (0.19-0.23) | -0.72 (-1.00--0.44) |
| Armenia | 15.00 (11.81-19.03) | -0.29 (-0.51--0.06) | 4.71 (4.01-5.74) | 0.69 (0.64-0.74) | 0.26 (0.21-0.30) | 0.14 (-0.37-0.65) |
| Australia | 45.85 (34.48-59.16) | 1.35 (1.00-1.71) | 19.22 (16.64-22.80) | 0.60 (0.39-0.82) | 0.86 (0.70-0.99) | 4.33 (3.39-5.28) |
| Austria | 28.61 (21.79-37.44) | -0.03 (-0.28-0.21) | 9.62 (8.26-11.61) | 0.27 (0.23-0.31) | 0.46 (0.38-0.51) | -0.34 (-1.03-0.36) |
| Azerbaijan | 10.20 (7.00-13.91) | -0.18 (-0.28--0.08) | 4.83 (4.15-5.78) | 0.13 (0.06-0.21) | 0.06 (0.03-0.11) | -1.46 (-1.52--1.39) |
| Bahrain | 9.72 (6.15-15.22) | -0.29 (-0.36--0.22) | 2.38 (2.04-2.88) | 0.95 (0.77-1.13) | 0.18 (0.09-0.39) | -1.09 (-1.35--0.83) |
| Bangladesh | 18.00 (13.24-24.45) | -0.48 (-0.51--0.44) | 5.38 (4.61-6.53) | 0.22 (0.13-0.30) | 0.38 (0.22-0.59) | -1.35 (-1.48--1.22) |
| Barbados | 19.56 (15.85-24.03) | -0.07 (-0.39-0.25) | 3.53 (2.96-4.54) | 0.62 (0.51-0.73) | 0.52 (0.41-0.65) | 0.30 (-0.13-0.75) |
| Belarus | 14.33 (11.12-18.41) | -0.09 (-0.21-0.03) | 3.83 (3.28-4.67) | 0.84 (0.79-0.90) | 0.29 (0.24-0.37) | 0.14 (-0.09-0.37) |
| Belgium | 31.80 (25.09-39.24) | 0.04 (-0.40-0.49) | 8.98 (7.63-10.65) | 0.92 (0.76-1.08) | 0.72 (0.60-0.81) | -0.54 (-1.56-0.49) |
| Belize | 12.61 (10.39-14.91) | -1.61 (-1.87--1.35) | 3.08 (2.62-3.83) | 0.77 (0.70-0.85) | 0.29 (0.25-0.33) | -2.01 (-2.42--1.60) |
| Benin | 30.59 (14.76-50.88) | 0.68 (0.53-0.83) | 1.44 (1.22-1.72) | -0.09 (-0.19-0.01) | 0.51 (0.22-0.90) | 0.61 (0.46-0.77) |
| Bermuda | 11.03 (8.49-14.20) | -3.75 (-4.16--3.33) | 3.06 (2.58-3.73) | 0.61 (0.57-0.66) | 0.20 (0.16-0.24) | -5.96 (-6.47--5.44) |
| Bhutan | 17.81 (12.74-24.51) | -0.21 (-0.26--0.16) | 5.74 (4.86-6.85) | 0.86 (0.78-0.94) | 0.40 (0.25-0.63) | -0.49 (-0.55--0.42) |
| Bolivia | 7.64 (5.25-11.41) | -1.40 (-1.47--1.33) | 1.69 (1.42-2.06) | 0.54 (0.47-0.61) | 0.21 (0.13-0.33) | -1.07 (-1.14--1.01) |
| Bosnia and Herzegovina | 17.25 (12.18-23.43) | -0.90 (-1.01--0.80) | 5.46 (4.62-6.58) | 1.02 (0.82-1.23) | 0.38 (0.23-0.60) | -1.35 (-1.58--1.12) |
| Botswana | 10.99 (7.51-15.42) | -0.37 (-0.74--0.01) | 2.05 (1.75-2.47) | 0.80 (0.76-0.85) | 0.32 (0.20-0.46) | -0.70 (-1.09--0.30) |
| Brazil | 17.29 (15.70-18.99) | 0.39 (0.16-0.62) | 2.92 (2.53-3.54) | 1.06 (0.59-1.52) | 0.51 (0.46-0.55) | 0.44 (0.14-0.74) |
| Brunei | 19.81 (14.18-28.63) | -0.50 (-0.70--0.29) | 0.90 (0.76-1.12) | 0.26 (0.21-0.31) | 0.90 (0.61-1.31) | -0.17 (-0.46-0.13) |
| Bulgaria | 14.06 (10.29-18.44) | 0.26 (0.13-0.39) | 7.25 (6.15-8.71) | 0.72 (0.65-0.79) | 0.15 (0.12-0.18) | 1.15 (0.64-1.67) |
| Burkina Faso | 27.70 (14.21-44.43) | 0.70 (0.46-0.95) | 1.33 (1.15-1.60) | -0.12 (-0.17--0.06) | 0.47 (0.22-0.75) | 0.62 (0.35-0.89) |
| Burundi | 9.97 (6.44-15.17) | -0.66 (-0.76--0.57) | 1.54 (1.30-1.86) | 0.59 (0.51-0.67) | 0.31 (0.16-0.53) | -0.72 (-0.84--0.59) |
| Cambodia | 10.02 (5.14-16.02) | -1.36 (-1.45--1.26) | 0.65 (0.55-0.80) | 0.76 (0.72-0.79) | 0.39 (0.20-0.64) | -1.41 (-1.51--1.32) |
| Cameroon | 28.43 (14.08-47.79) | 0.13 (0.04-0.22) | 1.72 (1.47-2.04) | 0.33 (0.18-0.47) | 0.48 (0.22-0.83) | 0.03 (-0.07-0.14) |
| Canada | 64.55 (45.86-87.53) | -0.68 (-1.01--0.36) | 28.24 (24.51-32.53) | -0.30 (-0.56--0.03) | 0.53 (0.46-0.59) | -2.07 (-2.69--1.44) |
| Cape Verde | 17.93 (10.89-28.46) | -1.90 (-1.98--1.82) | 1.42 (1.22-1.70) | 0.37 (0.30-0.45) | 0.28 (0.15-0.46) | -2.21 (-2.30--2.13) |
| Central African Republic | 10.47 (6.22-16.77) | 0.59 (0.47-0.70) | 1.65 (1.39-1.97) | 0.34 (0.32-0.36) | 0.31 (0.17-0.53) | 0.75 (0.59-0.92) |
| Chad | 33.12 (16.73-56.63) | 1.05 (0.94-1.16) | 1.27 (1.09-1.52) | -0.14 (-0.27--0.02) | 0.58 (0.28-1.02) | 1.04 (0.91-1.16) |
| Chile | 14.02 (11.09-17.84) | -0.85 (-1.05--0.65) | 4.44 (3.74-5.54) | 0.41 (0.30-0.53) | 0.25 (0.22-0.28) | -1.82 (-2.24--1.41) |
| China | 6.23 (4.38-9.44) | -3.77 (-4.06--3.49) | 1.41 (1.21-1.69) | 2.95 (2.42-3.49) | 0.25 (0.16-0.43) | -3.95 (-4.34--3.55) |
| Colombia | 7.80 (6.59-9.21) | -0.08 (-0.27-0.11) | 1.10 (0.91-1.34) | 0.44 (0.30-0.57) | 0.23 (0.19-0.27) | -0.11 (-0.38-0.16) |
| Comoros | 12.38 (8.65-17.44) | -0.44 (-0.57--0.30) | 1.81 (1.56-2.17) | 0.64 (0.44-0.85) | 0.37 (0.23-0.61) | -0.39 (-0.50--0.29) |
| Congo | 10.48 (6.80-16.64) | 0.39 (0.24-0.54) | 1.86 (1.61-2.21) | 0.86 (0.80-0.92) | 0.29 (0.16-0.50) | 0.44 (0.22-0.65) |
| Cook Islands | 2.25 (1.47-3.40) | -0.69 (-0.79--0.59) | 0.90 (0.76-1.08) | 0.77 (0.71-0.82) | 0.03 (0.01-0.07) | -1.49 (-1.64--1.34) |
| Costa Rica | 4.81 (4.19-5.45) | 0.09 (-0.12-0.31) | 0.62 (0.51-0.78) | 0.06 (-0.00-0.11) | 0.15 (0.13-0.17) | 0.17 (-0.07-0.41) |
| Cote d'Ivoire | 30.39 (14.62-51.55) | 1.41 (1.27-1.55) | 1.58 (1.36-1.89) | 0.06 (-0.09-0.21) | 0.51 (0.23-0.89) | 1.45 (1.29-1.61) |
| Croatia | 19.11 (14.62-24.85) | 0.08 (-0.33-0.49) | 7.87 (6.57-9.64) | 0.60 (0.10-1.11) | 0.30 (0.25-0.35) | 0.76 (0.36-1.16) |
| Cuba | 9.46 (7.48-11.89) | -1.95 (-2.17--1.74) | 2.79 (2.36-3.35) | 0.46 (0.44-0.49) | 0.16 (0.13-0.19) | -3.23 (-3.55--2.91) |
| Cyprus | 37.51 (28.39-48.09) | -1.88 (-2.07--1.68) | 8.14 (6.85-9.90) | 0.16 (-0.03-0.35) | 1.37 (0.88-1.94) | -3.17 (-3.48--2.86) |
| Czech Republic | 43.15 (32.41-56.64) | 0.47 (0.11-0.85) | 19.18 (16.58-22.33) | 0.94 (0.66-1.22) | 0.49 (0.40-0.58) | 0.67 (0.15-1.19) |
| Democratic Republic of the Congo | 8.48 (5.79-12.60) | -0.39 (-0.50--0.28) | 1.78 (1.50-2.17) | 1.10 (1.06-1.13) | 0.23 (0.13-0.41) | -0.10 (-0.14--0.06) |
| Denmark | 36.97 (27.13-48.34) | -0.05 (-0.43-0.33) | 16.92 (14.64-19.36) | -0.53 (-0.80--0.26) | 0.49 (0.42-0.55) | 0.61 (-0.21-1.45) |
| Djibouti | 9.98 (6.68-14.89) | -0.58 (-0.70--0.47) | 1.65 (1.41-1.99) | 0.38 (0.31-0.44) | 0.29 (0.17-0.51) | -0.67 (-0.78--0.56) |
| Dominica | 11.13 (8.18-15.03) | -0.34 (-0.43--0.25) | 3.32 (2.77-4.06) | 0.60 (0.45-0.75) | 0.22 (0.15-0.33) | -1.00 (-1.06--0.94) |
| Dominican Republic | 8.77 (6.40-12.04) | -0.77 (-0.89--0.65) | 2.86 (2.42-3.46) | 0.74 (0.67-0.81) | 0.14 (0.09-0.23) | -1.45 (-1.57--1.32) |
| Ecuador | 4.77 (3.85-5.99) | -2.06 (-2.39--1.74) | 1.48 (1.25-1.79) | 0.23 (0.17-0.28) | 0.11 (0.09-0.13) | -2.22 (-2.67--1.77) |
| Egypt | 16.53 (11.75-23.96) | -0.25 (-0.38--0.12) | 4.18 (3.51-5.21) | 0.81 (0.78-0.83) | 0.47 (0.29-0.81) | -0.38 (-0.62--0.14) |
| El Salvador | 5.35 (3.57-7.30) | -0.82 (-1.05--0.58) | 0.54 (0.47-0.66) | -0.44 (-0.49--0.39) | 0.17 (0.11-0.26) | -0.76 (-1.01--0.50) |
| Equatorial Guinea | 9.95 (5.59-16.35) | -0.66 (-0.88--0.43) | 2.17 (1.84-2.62) | 1.38 (1.14-1.62) | 0.26 (0.11-0.48) | -0.93 (-1.14--0.71) |
| Eritrea | 13.60 (8.85-19.54) | -0.07 (-0.16-0.02) | 1.70 (1.43-2.08) | 0.86 (0.79-0.93) | 0.42 (0.28-0.65) | -0.02 (-0.12-0.07) |
| Estonia | 11.67 (8.95-14.81) | -4.06 (-5.14--2.96) | 3.86 (3.32-4.73) | 1.21 (1.05-1.36) | 0.19 (0.16-0.22) | -6.59 (-8.28--4.88) |
| Ethiopia | 8.78 (5.88-12.97) | -0.93 (-1.08--0.78) | 1.58 (1.37-1.87) | 0.86 (0.78-0.95) | 0.26 (0.15-0.46) | -1.09 (-1.25--0.93) |
| Federated States of Micronesia | 3.05 (1.99-4.32) | -0.64 (-0.66--0.62) | 0.87 (0.72-1.07) | 0.60 (0.56-0.64) | 0.09 (0.05-0.15) | -0.76 (-0.79--0.73) |
| Fiji | 2.80 (1.96-3.91) | -0.46 (-0.60--0.32) | 0.93 (0.79-1.16) | 0.81 (0.70-0.92) | 0.07 (0.04-0.12) | -0.62 (-0.85--0.40) |
| Finland | 31.10 (22.34-42.05) | -0.48 (-0.92--0.04) | 13.82 (11.89-16.17) | -1.27 (-1.69--0.84) | 0.23 (0.19-0.26) | -0.44 (-0.85--0.04) |
| France | 41.64 (34.46-50.55) | 0.65 (0.41-0.88) | 10.10 (8.64-12.05) | 0.01 (-0.19-0.22) | 1.31 (1.06-1.51) | 1.19 (0.79-1.59) |
| Gabon | 8.95 (5.57-13.11) | 0.18 (0.02-0.35) | 1.99 (1.71-2.38) | 0.76 (0.73-0.79) | 0.23 (0.12-0.41) | 0.20 (-0.03-0.43) |
| Georgia | 14.31 (11.11-17.98) | -0.32 (-0.82-0.18) | 4.69 (4.02-5.62) | 0.12 (0.09-0.15) | 0.21 (0.17-0.26) | 0.38 (-0.81-1.59) |
| Germany | 72.16 (57.79-89.82) | 1.58 (1.23-1.94) | 17.19 (14.47-20.45) | 0.77 (0.41-1.13) | 1.90 (1.55-2.14) | 4.22 (3.60-4.84) |
| Ghana | 24.75 (12.74-43.50) | -0.23 (-0.30--0.15) | 1.55 (1.32-1.86) | 0.41 (0.28-0.53) | 0.40 (0.18-0.72) | -0.27 (-0.35--0.19) |
| Greece | 10.31 (8.13-12.90) | 0.86 (0.49-1.24) | 3.83 (3.28-4.62) | 0.92 (0.50-1.33) | 0.27 (0.22-0.30) | 1.18 (0.82-1.54) |
| Greenland | 51.20 (35.28-68.22) | -0.05 (-0.16-0.06) | 25.13 (22.04-29.11) | 0.50 (0.46-0.55) | 0.91 (0.29-1.48) | 0.51 (0.19-0.82) |
| Grenada | 24.32 (21.01-28.13) | -1.62 (-1.83--1.41) | 3.35 (2.87-4.04) | 0.90 (0.85-0.95) | 0.73 (0.63-0.83) | -1.49 (-1.75--1.23) |
| Guam | 2.29 (1.72-3.04) | 0.97 (0.37-1.58) | 0.94 (0.80-1.13) | 0.67 (0.62-0.73) | 0.03 (0.02-0.05) | 0.96 (-0.39-2.33) |
| Guatemala | 7.34 (6.35-8.41) | -0.90 (-1.34--0.45) | 0.63 (0.52-0.78) | 0.29 (0.22-0.36) | 0.24 (0.21-0.28) | -0.56 (-0.93--0.18) |
| Guinea | 30.11 (16.15-48.23) | 0.92 (0.79-1.06) | 1.42 (1.20-1.70) | -0.12 (-0.24--0.00) | 0.51 (0.25-0.83) | 0.92 (0.76-1.08) |
| Guinea-Bissau | 48.25 (27.13-77.13) | 0.89 (0.72-1.05) | 1.43 (1.22-1.72) | -0.07 (-0.20-0.05) | 0.85 (0.46-1.40) | 0.88 (0.72-1.05) |
| Guyana | 30.32 (23.40-38.23) | -0.52 (-0.83--0.21) | 3.22 (2.72-4.01) | 0.78 (0.72-0.85) | 0.83 (0.63-1.07) | -0.68 (-1.08--0.28) |
| Haiti | 31.88 (15.48-55.01) | -0.45 (-0.50--0.40) | 2.80 (2.32-3.44) | 0.58 (0.50-0.65) | 0.97 (0.41-1.86) | -0.46 (-0.53--0.40) |
| Honduras | 11.92 (6.89-19.84) | -1.02 (-1.09--0.95) | 0.62 (0.52-0.77) | 0.18 (0.12-0.25) | 0.40 (0.24-0.71) | -0.60 (-0.69--0.52) |
| Hungary | 40.08 (31.05-51.03) | -0.11 (-0.42-0.21) | 14.63 (12.64-17.16) | 0.85 (0.47-1.23) | 0.58 (0.49-0.68) | 0.18 (-0.28-0.63) |
| Iceland | 45.85 (35.99-58.41) | -0.22 (-0.41--0.03) | 16.59 (14.33-19.28) | -0.53 (-0.66--0.40) | 0.89 (0.73-1.03) | 1.36 (0.86-1.86) |
| India | 15.16 (10.95-21.19) | -0.50 (-0.60--0.40) | 5.84 (5.06-6.98) | 0.75 (0.65-0.85) | 0.28 (0.17-0.47) | -1.26 (-1.41--1.11) |
| Indonesia | 8.07 (4.97-10.94) | -2.12 (-2.28--1.95) | 0.73 (0.62-0.88) | 0.17 (0.11-0.23) | 0.37 (0.21-0.52) | -1.90 (-2.07--1.74) |
| Iran | 7.99 (5.55-10.93) | 0.30 (0.08-0.51) | 2.81 (2.40-3.41) | 0.26 (0.00-0.52) | 0.11 (0.04-0.17) | 0.08 (-0.32-0.47) |
| Iraq | 12.27 (8.77-17.14) | -0.67 (-0.77--0.56) | 2.75 (2.33-3.33) | 1.17 (1.10-1.24) | 0.26 (0.15-0.45) | -1.04 (-1.11--0.96) |
| Ireland | 38.11 (29.14-48.80) | 0.20 (-0.11-0.51) | 13.57 (11.63-16.28) | 0.09 (-0.04-0.22) | 0.71 (0.56-0.85) | -0.19 (-0.79-0.42) |
| Israel | 25.83 (18.59-34.69) | -0.34 (-0.83-0.16) | 12.72 (10.74-15.45) | -0.01 (-0.47-0.46) | 0.33 (0.26-0.38) | 0.51 (-0.13-1.15) |
| Italy | 27.46 (22.85-32.91) | 0.78 (0.57-1.00) | 7.44 (6.45-8.81) | -0.42 (-0.55--0.28) | 0.84 (0.68-0.96) | 3.42 (2.92-3.93) |
| Jamaica | 11.46 (9.10-14.59) | 0.17 (-0.06-0.41) | 2.60 (2.22-3.19) | 0.32 (0.22-0.42) | 0.21 (0.16-0.27) | 0.22 (-0.20-0.65) |
| Japan | 5.65 (4.15-7.57) | -1.15 (-1.45--0.84) | 1.90 (1.64-2.26) | -0.02 (-0.41-0.37) | 0.06 (0.05-0.07) | -3.44 (-4.15--2.72) |
| Jordan | 14.22 (10.29-18.79) | -0.16 (-0.36-0.04) | 3.21 (2.71-3.93) | 0.90 (0.75-1.05) | 0.31 (0.18-0.45) | -0.31 (-0.74-0.13) |
| Kazakhstan | 22.71 (18.39-27.96) | -0.50 (-0.72--0.29) | 5.25 (4.48-6.39) | 0.60 (0.45-0.74) | 0.42 (0.32-0.54) | -0.69 (-1.01--0.36) |
| Kenya | 11.09 (6.93-16.33) | 0.21 (0.12-0.31) | 1.71 (1.48-2.02) | 0.87 (0.78-0.96) | 0.35 (0.19-0.56) | 0.38 (0.24-0.51) |
| Kiribati | 3.23 (2.01-4.83) | 0.15 (0.08-0.23) | 0.80 (0.68-0.98) | 0.56 (0.52-0.60) | 0.10 (0.05-0.18) | 0.29 (0.19-0.40) |
| Kuwait | 11.27 (8.50-14.51) | 0.41 (-0.35-1.18) | 3.61 (3.06-4.41) | -0.19 (-0.52-0.14) | 0.16 (0.13-0.19) | 6.37 (3.59-9.23) |
| Kyrgyzstan | 13.13 (10.15-17.19) | -3.05 (-3.61--2.48) | 4.43 (3.81-5.37) | 0.15 (0.13-0.17) | 0.16 (0.12-0.20) | -5.07 (-6.13--4.00) |
| Laos | 5.12 (2.69-8.29) | -1.30 (-1.36--1.24) | 0.65 (0.55-0.79) | 0.74 (0.70-0.78) | 0.18 (0.08-0.32) | -1.67 (-1.75--1.58) |
| Latvia | 14.06 (11.13-17.50) | -3.63 (-4.79--2.46) | 4.08 (3.42-4.91) | 0.73 (0.64-0.82) | 0.28 (0.24-0.33) | -5.33 (-7.08--3.56) |
| Lebanon | 20.60 (15.33-26.95) | -0.31 (-0.36--0.25) | 7.18 (6.14-8.69) | 0.87 (0.76-0.98) | 0.26 (0.17-0.39) | -1.40 (-1.46--1.33) |
| Lesotho | 14.24 (9.39-20.94) | 1.33 (1.01-1.67) | 1.75 (1.49-2.10) | 0.42 (0.35-0.49) | 0.45 (0.27-0.73) | 1.30 (0.92-1.69) |
| Liberia | 27.96 (14.71-49.75) | 0.82 (0.69-0.95) | 1.44 (1.24-1.74) | -0.30 (-0.53--0.06) | 0.47 (0.23-0.87) | 0.78 (0.63-0.93) |
| Libya | 11.94 (7.39-22.56) | 1.68 (1.44-1.92) | 3.00 (2.53-3.66) | 2.52 (2.37-2.67) | 0.27 (0.10-0.68) | 2.04 (1.60-2.47) |
| Lithuania | 13.47 (9.95-17.53) | -2.16 (-2.98--1.34) | 5.48 (4.56-6.83) | 1.28 (0.90-1.67) | 0.20 (0.17-0.23) | -4.58 (-6.28--2.85) |
| Luxembourg | 39.30 (30.91-49.24) | 0.54 (0.28-0.80) | 11.35 (9.81-13.71) | -0.30 (-0.39--0.21) | 1.00 (0.84-1.13) | 1.05 (0.52-1.59) |
| Macedonia | 13.34 (9.71-18.01) | 0.08 (0.02-0.13) | 7.06 (6.03-8.55) | 0.75 (0.67-0.83) | 0.16 (0.08-0.23) | 0.87 (0.54-1.21) |
| Madagascar | 12.06 (7.54-19.21) | -0.09 (-0.14--0.04) | 1.70 (1.46-2.03) | 0.70 (0.64-0.77) | 0.36 (0.19-0.63) | -0.12 (-0.16--0.08) |
| Malawi | 10.96 (7.08-16.18) | -0.14 (-0.26--0.02) | 1.81 (1.55-2.19) | 1.02 (0.92-1.12) | 0.33 (0.19-0.53) | -0.11 (-0.26-0.04) |
| Malaysia | 2.60 (1.91-3.41) | 0.61 (0.34-0.89) | 0.78 (0.67-0.92) | 1.52 (1.27-1.77) | 0.05 (0.03-0.07) | -0.27 (-0.64-0.10) |
| Maldives | 3.69 (2.69-4.89) | -2.01 (-2.17--1.86) | 0.64 (0.54-0.78) | 1.09 (0.97-1.22) | 0.13 (0.08-0.18) | -2.39 (-2.58--2.20) |
| Mali | 46.73 (26.67-72.50) | 0.67 (0.58-0.76) | 1.35 (1.17-1.60) | -0.10 (-0.20-0.00) | 0.83 (0.46-1.29) | 0.63 (0.53-0.72) |
| Malta | 26.66 (21.08-33.58) | 0.47 (0.25-0.69) | 7.10 (6.00-8.69) | -0.14 (-0.25--0.02) | 0.63 (0.52-0.71) | 0.80 (0.28-1.32) |
| Marshall Islands | 2.82 (1.89-4.26) | -0.59 (-0.70--0.48) | 0.83 (0.69-1.00) | 0.58 (0.56-0.61) | 0.08 (0.04-0.14) | -0.97 (-1.10--0.83) |
| Mauritania | 25.33 (14.86-41.99) | 0.71 (0.51-0.90) | 1.44 (1.23-1.74) | -0.16 (-0.43-0.10) | 0.42 (0.22-0.74) | 0.69 (0.49-0.89) |
| Mauritius | 10.33 (9.40-11.29) | 2.28 (0.82-3.76) | 0.68 (0.57-0.83) | 0.50 (0.47-0.52) | 0.41 (0.35-0.45) | 2.76 (0.96-4.59) |
| Mexico | 11.21 (9.62-12.86) | 0.91 (0.60-1.22) | 0.19 (0.16-0.24) | 0.08 (-0.19-0.35) | 0.46 (0.39-0.52) | 0.64 (0.32-0.97) |
| Moldova | 10.94 (8.65-13.62) | -3.27 (-4.45--2.07) | 4.01 (3.38-4.83) | 0.48 (0.33-0.63) | 0.21 (0.17-0.24) | -5.31 (-7.28--3.29) |
| Monaco | 29.84 (21.90-40.18) | 0.08 (0.06-0.11) | 11.84 (10.07-14.15) | -0.16 (-0.21--0.10) | 0.37 (0.23-0.54) | 0.06 (-0.04-0.17) |
| Mongolia | 14.41 (10.01-20.34) | -2.29 (-2.44--2.13) | 5.18 (4.38-6.24) | 0.67 (0.64-0.70) | 0.28 (0.14-0.49) | -2.38 (-2.57--2.20) |
| Montenegro | 19.46 (14.28-26.25) | 0.02 (-0.06-0.10) | 6.20 (5.26-7.54) | 0.25 (0.15-0.35) | 0.33 (0.19-0.50) | 0.41 (0.32-0.51) |
| Morocco | 10.01 (6.53-16.54) | 0.41 (0.26-0.56) | 2.98 (2.54-3.67) | 0.76 (0.72-0.80) | 0.18 (0.09-0.40) | 0.39 (0.20-0.58) |
| Mozambique | 11.07 (6.80-17.27) | 0.43 (0.37-0.48) | 1.62 (1.39-1.96) | 0.92 (0.85-0.98) | 0.35 (0.19-0.62) | 0.59 (0.51-0.67) |
| Myanmar | 4.07 (2.57-6.03) | -2.17 (-2.32--2.02) | 0.70 (0.58-0.86) | 0.85 (0.80-0.90) | 0.11 (0.06-0.16) | -2.73 (-2.92--2.55) |
| Namibia | 13.58 (8.50-21.40) | -0.59 (-0.65--0.53) | 1.71 (1.47-2.06) | 0.19 (0.10-0.29) | 0.42 (0.23-0.69) | -0.63 (-0.75--0.52) |
| Nauru | 3.10 (1.91-4.78) | -0.48 (-0.59--0.37) | 0.93 (0.78-1.13) | 0.42 (0.32-0.53) | 0.08 (0.04-0.16) | -0.78 (-0.96--0.60) |
| Nepal | 17.21 (12.37-23.39) | -0.96 (-1.06--0.87) | 5.58 (4.68-6.88) | 0.77 (0.67-0.87) | 0.38 (0.24-0.60) | -1.42 (-1.56--1.27) |
| Netherlands | 78.73 (63.52-96.62) | 0.57 (0.05-1.08) | 22.54 (19.53-26.30) | 1.52 (0.86-2.19) | 2.33 (1.90-2.60) | 0.79 (0.32-1.26) |
| New Zealand | 36.37 (26.04-48.55) | -0.19 (-0.49-0.12) | 25.24 (21.59-29.88) | 0.03 (-0.06-0.12) | 0.38 (0.32-0.43) | 0.05 (-0.94-1.05) |
| Nicaragua | 3.81 (2.57-5.06) | -0.24 (-0.38--0.10) | 0.58 (0.48-0.71) | 0.12 (-0.02-0.25) | 0.11 (0.07-0.16) | 0.15 (-0.09-0.38) |
| Niger | 29.41 (14.15-56.05) | 0.32 (0.17-0.46) | 1.18 (1.00-1.41) | -0.22 (-0.33--0.12) | 0.50 (0.22-0.99) | 0.31 (0.16-0.46) |
| Nigeria | 22.22 (10.39-37.70) | 0.50 (0.43-0.58) | 1.52 (1.33-1.79) | 0.08 (-0.04-0.19) | 0.36 (0.14-0.63) | 0.41 (0.33-0.50) |
| Niue | 5.39 (2.81-10.60) | 0.13 (-0.40-0.66) | 0.86 (0.73-1.05) | 0.81 (0.75-0.88) | 0.10 (0.05-0.18) | -0.43 (-0.71--0.15) |
| North Korea | 10.23 (6.28-16.54) | -0.80 (-0.88--0.72) | 0.94 (0.80-1.14) | 0.50 (0.45-0.55) | 0.45 (0.25-0.79) | -0.88 (-0.95--0.80) |
| Northern Mariana Islands | 1.57 (1.08-2.17) | -0.45 (-0.55--0.35) | 0.92 (0.77-1.12) | 0.38 (0.32-0.43) | 0.01 (0.00-0.01) | -1.51 (-2.31--0.71) |
| Norway | 42.62 (31.14-56.40) | -0.04 (-0.23-0.14) | 19.13 (16.97-21.59) | 0.69 (0.55-0.83) | 0.37 (0.31-0.40) | -0.47 (-1.05-0.11) |
| Oman | 9.41 (6.17-13.72) | 0.26 (0.10-0.41) | 3.19 (2.71-3.87) | 1.28 (1.20-1.37) | 0.10 (0.04-0.25) | -0.43 (-0.70--0.16) |
| Pakistan | 21.19 (14.62-29.57) | -0.51 (-0.60--0.42) | 5.93 (5.15-7.14) | 0.67 (0.60-0.74) | 0.60 (0.36-0.90) | -0.82 (-0.95--0.70) |
| Palau | 3.36 (2.21-4.80) | -0.11 (-0.14--0.09) | 0.90 (0.78-1.10) | 0.51 (0.46-0.56) | 0.10 (0.05-0.17) | 0.13 (0.07-0.19) |
| Palestine | 16.35 (12.55-21.99) | -2.06 (-2.25--1.86) | 3.07 (2.57-3.82) | 1.28 (1.22-1.34) | 0.49 (0.33-0.74) | -2.61 (-2.74--2.49) |
| Panama | 15.27 (12.36-18.06) | 0.52 (0.30-0.73) | 0.92 (0.75-1.15) | 0.15 (0.12-0.19) | 0.54 (0.42-0.64) | 0.79 (0.55-1.03) |
| Papua New Guinea | 1.95 (1.41-2.67) | -0.91 (-1.08--0.75) | 0.71 (0.59-0.89) | 0.50 (0.47-0.53) | 0.04 (0.02-0.07) | -0.85 (-0.98--0.72) |
| Paraguay | 6.22 (4.44-8.33) | 0.28 (0.13-0.43) | 1.63 (1.38-1.95) | 0.19 (0.05-0.34) | 0.15 (0.09-0.21) | 1.00 (0.85-1.15) |
| Peru | 5.78 (4.16-8.19) | -3.25 (-3.73--2.76) | 1.60 (1.37-1.97) | 0.68 (0.60-0.77) | 0.13 (0.08-0.21) | -2.89 (-3.26--2.51) |
| Philippines | 4.22 (2.98-5.95) | -1.41 (-1.61--1.21) | 0.50 (0.42-0.62) | 0.46 (0.41-0.51) | 0.15 (0.09-0.23) | -1.79 (-2.03--1.54) |
| Poland | 14.09 (12.27-16.28) | 0.06 (-0.26-0.38) | 2.58 (2.22-3.09) | 0.48 (0.36-0.60) | 0.42 (0.37-0.47) | 0.31 (-0.13-0.75) |
| Portugal | 27.74 (22.97-33.09) | 0.67 (0.32-1.03) | 7.99 (6.77-9.56) | 0.80 (0.53-1.07) | 0.93 (0.76-1.05) | 1.59 (1.01-2.18) |
| Puerto Rico | 11.99 (9.74-14.76) | -2.72 (-2.97--2.48) | 2.79 (2.38-3.40) | 0.69 (0.61-0.76) | 0.26 (0.21-0.31) | -4.13 (-4.49--3.76) |
| Qatar | 17.81 (12.96-23.46) | -1.72 (-1.89--1.55) | 3.50 (2.94-4.26) | 0.84 (0.83-0.85) | 0.50 (0.29-0.76) | -3.05 (-3.42--2.68) |
| Romania | 5.99 (4.82-7.30) | 0.18 (-0.25-0.61) | 2.13 (1.81-2.59) | 1.35 (0.55-2.15) | 0.12 (0.10-0.15) | 0.64 (0.28-1.01) |
| Russian Federation | 19.99 (17.56-22.83) | -0.83 (-1.40--0.25) | 3.92 (3.40-4.76) | 0.48 (0.45-0.50) | 0.57 (0.51-0.63) | -0.77 (-1.75-0.22) |
| Rwanda | 11.49 (6.98-18.31) | -0.56 (-0.71--0.42) | 1.78 (1.52-2.19) | 0.98 (0.89-1.07) | 0.36 (0.19-0.64) | -0.55 (-0.71--0.39) |
| Saint Kitts and Nevis | 10.52 (8.40-13.11) | -2.44 (-2.68--2.20) | 3.45 (2.91-4.21) | 0.56 (0.44-0.68) | 0.21 (0.18-0.25) | -3.17 (-3.47--2.87) |
| Saint Lucia | 19.60 (16.00-23.35) | -2.30 (-2.71--1.89) | 2.94 (2.46-3.61) | 0.45 (0.41-0.50) | 0.56 (0.44-0.67) | -3.23 (-3.83--2.62) |
| Saint Vincent and the Grenadines | 25.74 (21.77-29.90) | -1.24 (-1.57--0.90) | 3.14 (2.70-3.78) | 0.57 (0.50-0.65) | 0.72 (0.61-0.83) | -1.44 (-1.86--1.03) |
| Samoa | 2.92 (1.97-4.04) | -0.10 (-0.14--0.06) | 0.74 (0.63-0.90) | 0.49 (0.44-0.54) | 0.08 (0.05-0.14) | -0.05 (-0.09--0.01) |
| San Marino | 32.75 (22.46-47.22) | 0.01 (-0.10-0.12) | 12.65 (10.59-15.43) | -0.21 (-0.30--0.12) | 0.20 (0.10-0.34) | -1.50 (-1.84--1.16) |
| Sao Tome and Principe | 21.12 (9.36-46.37) | -0.06 (-0.37-0.25) | 1.41 (1.20-1.73) | 0.38 (0.23-0.53) | 0.34 (0.12-0.80) | -0.11 (-0.44-0.23) |
| Saudi Arabia | 8.81 (6.22-12.49) | 0.06 (-0.14-0.27) | 2.06 (1.76-2.50) | -0.57 (-0.73--0.40) | 0.14 (0.07-0.22) | -0.93 (-1.27--0.58) |
| Senegal | 31.68 (18.19-49.46) | 0.00 (-0.14-0.15) | 1.52 (1.28-1.82) | 0.58 (0.33-0.83) | 0.54 (0.29-0.88) | -0.06 (-0.22-0.10) |
| Serbia | 24.29 (17.72-31.41) | -0.29 (-0.41--0.17) | 7.07 (5.94-8.75) | 0.61 (0.52-0.69) | 0.57 (0.36-0.76) | -0.31 (-0.52--0.10) |
| Seychelles | 4.10 (2.78-5.77) | -0.87 (-0.98--0.76) | 0.68 (0.58-0.84) | 0.43 (0.37-0.48) | 0.14 (0.08-0.21) | -1.04 (-1.24--0.83) |
| Sierra Leone | 30.18 (16.79-46.35) | 1.79 (1.68-1.89) | 1.45 (1.23-1.77) | -0.17 (-0.32--0.03) | 0.51 (0.27-0.80) | 1.86 (1.75-1.97) |
| Singapore | 2.08 (1.41-2.90) | -2.35 (-2.53--2.18) | 0.76 (0.64-0.91) | 0.76 (0.60-0.91) | 0.01 (0.01-0.02) | -7.15 (-7.42--6.88) |
| Slovakia | 23.64 (17.17-30.52) | 0.02 (-0.11-0.14) | 6.95 (5.94-8.43) | 1.10 (0.97-1.23) | 0.52 (0.30-0.70) | 0.43 (0.17-0.70) |
| Slovenia | 23.00 (17.74-30.17) | -0.43 (-0.74--0.13) | 10.58 (9.05-12.58) | 0.71 (0.54-0.88) | 0.32 (0.27-0.36) | -0.68 (-1.27--0.09) |
| Solomon Islands | 2.02 (1.39-2.72) | 0.11 (0.05-0.17) | 0.73 (0.62-0.89) | 0.53 (0.51-0.56) | 0.05 (0.02-0.08) | 0.16 (0.06-0.25) |
| Somalia | 9.33 (5.81-15.96) | -0.45 (-0.54--0.36) | 1.24 (1.06-1.50) | 0.37 (0.33-0.42) | 0.28 (0.16-0.51) | -0.41 (-0.49--0.33) |
| South Africa | 11.18 (7.95-14.52) | 0.10 (-0.27-0.47) | 1.68 (1.46-2.00) | 0.22 (0.13-0.32) | 0.31 (0.19-0.43) | 0.38 (-0.04-0.80) |
| South Korea | 12.23 (8.38-18.09) | -2.86 (-2.99--2.74) | 4.15 (3.57-4.98) | 1.65 (0.91-2.39) | 0.24 (0.13-0.57) | -6.55 (-6.99--6.11) |
| South Sudan | 11.09 (6.72-16.60) | -0.20 (-0.30--0.09) | 1.51 (1.28-1.83) | 0.51 (0.44-0.57) | 0.33 (0.19-0.52) | -0.16 (-0.24--0.09) |
| Spain | 21.68 (15.58-28.87) | -0.36 (-0.79-0.07) | 8.79 (7.48-10.53) | 0.47 (-0.18-1.12) | 0.26 (0.21-0.30) | -1.33 (-1.70--0.95) |
| Sri Lanka | 2.06 (1.46-2.82) | -0.93 (-1.09--0.76) | 0.91 (0.77-1.11) | 1.13 (0.83-1.43) | 0.02 (0.01-0.04) | -3.52 (-3.68--3.35) |
| Sudan | 10.69 (7.18-16.40) | 0.95 (0.80-1.11) | 3.00 (2.52-3.75) | 1.02 (0.97-1.08) | 0.20 (0.10-0.37) | 1.05 (0.87-1.23) |
| Suriname | 20.84 (15.12-29.55) | -1.20 (-1.35--1.06) | 3.00 (2.53-3.73) | 0.58 (0.53-0.64) | 0.54 (0.34-0.85) | -1.39 (-1.52--1.25) |
| Swaziland | 13.81 (8.45-20.96) | 0.35 (0.11-0.59) | 1.90 (1.63-2.28) | 0.45 (0.39-0.52) | 0.42 (0.25-0.68) | 0.27 (-0.02-0.55) |
| Sweden | 39.58 (28.16-52.18) | -1.08 (-1.42--0.73) | 19.32 (17.38-21.43) | 0.13 (0.07-0.20) | 0.39 (0.32-0.45) | -4.30 (-5.75--2.83) |
| Switzerland | 32.64 (24.19-42.86) | -0.12 (-0.35-0.11) | 11.28 (9.68-13.65) | 0.17 (0.15-0.19) | 0.56 (0.44-0.63) | 0.11 (-0.68-0.90) |
| Syria | 15.84 (10.66-22.03) | -0.34 (-0.38--0.30) | 3.15 (2.67-3.89) | 0.83 (0.60-1.06) | 0.45 (0.26-0.70) | -0.46 (-0.58--0.34) |
| Taiwan | 7.29 (6.29-8.19) | -3.50 (-4.13--2.86) | 0.84 (0.71-1.04) | 2.04 (1.36-2.73) | 0.39 (0.31-0.44) | -3.65 (-4.33--2.97) |
| Tajikistan | 16.49 (10.77-25.45) | -0.63 (-0.80--0.46) | 4.59 (3.92-5.57) | 0.14 (0.04-0.23) | 0.23 (0.13-0.39) | -1.03 (-1.15--0.90) |
| Tanzania | 11.01 (7.02-16.70) | 0.38 (0.26-0.50) | 1.70 (1.46-2.06) | 0.61 (0.47-0.74) | 0.31 (0.17-0.52) | 0.42 (0.27-0.57) |
| Thailand | 2.69 (1.83-3.65) | 0.08 (-0.04-0.21) | 0.60 (0.51-0.73) | 0.64 (0.60-0.69) | 0.08 (0.04-0.12) | 0.23 (-0.00-0.47) |
| The Bahamas | 17.75 (14.51-21.72) | -1.70 (-1.96--1.45) | 3.50 (2.96-4.31) | 0.83 (0.73-0.93) | 0.42 (0.34-0.52) | -1.94 (-2.32--1.56) |
| The Gambia | 43.97 (23.67-72.46) | 0.92 (0.69-1.15) | 1.48 (1.26-1.84) | 0.62 (0.49-0.74) | 0.77 (0.41-1.32) | 0.90 (0.66-1.15) |
| Timor-Leste | 6.66 (3.15-11.89) | -0.75 (-0.95--0.55) | 0.64 (0.54-0.79) | 0.92 (0.84-0.99) | 0.25 (0.10-0.49) | -0.86 (-1.03--0.69) |
| Togo | 42.68 (21.52-70.94) | 0.52 (0.45-0.59) | 1.62 (1.38-1.92) | 0.43 (0.34-0.53) | 0.75 (0.35-1.29) | 0.51 (0.43-0.59) |
| Tokelau | 4.67 (3.04-7.60) | -0.38 (-0.79-0.03) | 0.85 (0.73-1.05) | 0.18 (0.09-0.27) | 0.10 (0.05-0.15) | -1.31 (-1.53--1.08) |
| Tonga | 3.78 (2.43-6.00) | -0.15 (-0.21--0.08) | 0.75 (0.64-0.91) | 0.71 (0.67-0.76) | 0.12 (0.06-0.22) | -0.15 (-0.23--0.07) |
| Trinidad and Tobago | 14.80 (11.79-18.43) | -1.03 (-1.25--0.80) | 3.26 (2.73-3.91) | 0.23 (0.07-0.38) | 0.30 (0.23-0.38) | -1.96 (-2.29--1.63) |
| Tunisia | 9.20 (6.15-13.85) | 0.44 (0.32-0.56) | 2.66 (2.25-3.19) | 0.64 (0.60-0.68) | 0.12 (0.06-0.28) | -0.10 (-0.24-0.04) |
| Turkey | 14.16 (10.66-18.39) | -0.81 (-0.89--0.74) | 2.70 (2.30-3.19) | 0.14 (-0.08-0.35) | 0.37 (0.24-0.52) | -0.59 (-0.84--0.34) |
| Turkmenistan | 17.37 (12.54-24.02) | -0.40 (-0.48--0.31) | 5.52 (4.76-6.66) | 0.49 (0.41-0.57) | 0.27 (0.18-0.44) | -0.69 (-0.87--0.51) |
| Tuvalu | 3.04 (2.03-4.37) | -0.72 (-0.77--0.67) | 0.86 (0.74-1.05) | 0.87 (0.82-0.92) | 0.09 (0.05-0.16) | -0.84 (-0.88--0.80) |
| Uganda | 7.45 (5.25-11.01) | 0.12 (-0.01-0.24) | 1.73 (1.48-2.05) | 0.81 (0.72-0.90) | 0.21 (0.13-0.36) | -0.10 (-0.27-0.07) |
| Ukraine | 13.36 (9.60-17.31) | -0.42 (-0.56--0.29) | 4.60 (3.92-5.65) | 0.81 (0.63-0.99) | 0.26 (0.17-0.37) | -0.38 (-0.57--0.19) |
| United Arab Emirates | 12.51 (8.94-17.45) | 1.21 (0.97-1.45) | 4.00 (3.36-4.95) | 1.06 (0.90-1.21) | 0.28 (0.15-0.49) | 4.62 (3.86-5.39) |
| United Kingdom | 43.19 (35.92-51.58) | 0.01 (-0.30-0.32) | 15.72 (13.70-18.23) | 0.17 (-0.02-0.35) | 1.16 (1.01-1.24) | 0.57 (0.01-1.14) |
| United States | 49.57 (41.28-59.80) | 0.90 (0.70-1.11) | 17.42 (15.31-20.05) | 0.67 (0.55-0.79) | 1.08 (0.94-1.16) | 2.05 (1.61-2.49) |
| Uruguay | 17.11 (14.28-20.84) | -0.59 (-0.67--0.51) | 4.64 (3.90-5.79) | 0.85 (0.77-0.93) | 0.38 (0.34-0.41) | -0.69 (-0.84--0.54) |
| Uzbekistan | 15.04 (11.87-18.78) | -0.23 (-0.50-0.04) | 5.35 (4.62-6.38) | 0.73 (0.69-0.78) | 0.21 (0.16-0.27) | -0.06 (-0.59-0.48) |
| Vanuatu | 3.42 (1.87-5.43) | -0.04 (-0.16-0.09) | 0.75 (0.63-0.91) | 0.56 (0.54-0.58) | 0.11 (0.04-0.20) | -0.12 (-0.27-0.03) |
| Venezuela | 5.31 (3.95-6.94) | -0.25 (-0.51-0.02) | 0.95 (0.79-1.20) | -0.08 (-0.28-0.12) | 0.15 (0.10-0.20) | -0.27 (-0.56-0.01) |
| Vietnam | 3.20 (2.06-4.78) | -1.40 (-1.53--1.28) | 0.61 (0.51-0.74) | 0.83 (0.76-0.89) | 0.11 (0.06-0.18) | -1.96 (-2.10--1.82) |
| Virgin Islands, U.S. | 17.20 (10.93-25.81) | -2.01 (-2.23--1.79) | 3.22 (2.70-3.96) | 0.63 (0.57-0.70) | 0.37 (0.20-0.61) | -3.29 (-3.52--3.05) |
| Yemen | 9.70 (6.17-15.56) | 0.93 (0.75-1.12) | 2.81 (2.38-3.46) | 0.84 (0.82-0.87) | 0.20 (0.10-0.43) | 0.96 (0.79-1.13) |
| Zambia | 11.13 (7.09-16.54) | -0.12 (-0.21--0.04) | 1.99 (1.70-2.36) | 0.95 (0.84-1.06) | 0.31 (0.17-0.54) | -0.13 (-0.25--0.01) |
| Zimbabwe | 13.07 (6.96-21.52) | 1.27 (0.90-1.64) | 1.66 (1.42-2.04) | 0.29 (0.04-0.54) | 0.42 (0.21-0.73) | 1.31 (0.84-1.77) |
